# Human Cyclophilins—An Emerging Class of Drug Targets

**DOI:** 10.1002/med.70021

**Published:** 2025-10-29

**Authors:** Katarina Jurkova, Hana Navratilova, Kamil Musilek, Ondrej Benek

**Affiliations:** ^1^ Department of Chemistry Faculty of Science University of Hradec Kralove Hradec Kralove Czech Republic; ^2^ University Hospital Hradec Kralove, Biomedical Research Centre Hradec Kralove Czech Republic

**Keywords:** cyclophilin (Cyp), cyclosporine A (CsA), drug target, enzyme inhibition, peptidyl‐prolyl *cis‐trans* isomerase (PPIase)

## Abstract

Cyclophilins are a family of enzymes with peptidyl‐prolyl isomerase activity found in all cells of all organisms. To date, 17 cyclophilin isoforms have been identified in the human body, participating in diverse biological processes. Consequently, cyclophilins have emerged as promising targets for drug development to address a wide array of human diseases. This review describes the structural characteristics of individual cyclophilin isoforms and explores the roles that they play in human health and diseases, such as in viral infections, Alzheimer's disease, Parkinson's disease, cardiovascular diseases, or cancer. Additionally, the review addresses inhibition of cyclophilins, particularly focusing on the development of selective small‐molecule inhibitors of individual cyclophilins, which possess a significant potential as novel therapeutics.

**Abbreviations**
AA: amino acid
AAA: abdominal aortic aneurysm
AD: Alzheimer's disease
AIDS: acquired immunodeficiency syndrome
ALS: amyotrophic lateral sclerosis
AngII: angiotensin II
ANT: adenine nucleotide translocase
AP: acute pancreatitis
APP: amyloid beta precursor protein
AR: androgen receptor
ATP: adenosine triphosphate
Aβ: amyloid beta
BBB: blood‐brain barrier
CAD: coronary artery disease
CLD: cyclophilin‐like domain
CsA: cyclosporine A
Cyp: cyclophilin
CypCAP: cyclophilin C associated protein
ER: endoplasmic reticulum
ERK1/2: extracellular signal‐regulated kinase 1/2
FKBP: FK‐506 binding protein
GTP: guanosine triphosphate
HBV: hepatitis B virus
HCC: hepatocellular carcinoma
HCV: hepatitis C virus
HIV‐1: human immunodeficiency virus type 1
HNK: honokiol
Hsp90: heat shock protein 90
IC_50_
: half‐maximal inhibitory concentration
IFN‐I: interferon type 1
IL‐2: interleukin‐2
IMM: inner mitochondrial membrane
IRI: ischemia‐reperfusion injury
ITC: isothermal titration calorimetry
JEV: Japanese encephalitis virus
JNK: c‐Jun N‐terminal protein kinase
kDa: kilodalton
lncRNA: long noncoding RNA
MERS: Middle East respiratory syndrome
MHC: major histocompatibility complex class molecules
miRNA: MicroRNA, small noncoding RNA
MLL: mixed lineage leukemia
MMP: matrix metalloproteinase
MPP+: 1‐methyl‐4‐phenylpyridinium
mPTP: mitochondrial permeability transition pore
MSC: major spliceosome complex
NAFLD: nonalcoholic fatty liver disease
NASH: nonalcoholic steatohepatitis
NF‐AT: nuclear factor of activated T‐cells
NK: natural killer
NMR: nuclear magnetic resonance
NP: nucleoprotein
NPC: nuclear pore complex
NS: nonstructural
NTD: N‐terminal domain
ORFV: Orf virus
PCa: prostate cancer
PD: Parkinson's disease
PDB: Protein Data Bank
PMO: postmenopausal osteoporosis
PPIase: peptidyl‐prolyl isomerase
PPIL: peptidyl‐prolyl isomerase like
PPWD1: peptidyl‐prolyl isomerase containing WD40 repeat
PRL: prolactin
RANBP2: Ran‐binding protein 2
RBD: Ran‐binding domain
RIG‐I: retinoic acid‐inducible protein I
RNP: ribonucleoprotein
ROS: reactive oxygen species
RRM: RNA recognition motif
SARS: severe acute respiratory syndrome
SCLC: small cell lung cancer
SDCCAG‐10: serological defined colon cancer antigen 10
SfA: Sanglifehrin A
SKIP: SKI‐interacting protein
snRNP: small nuclear ribonucleoprotein particle
SOD1: superoxide dismutase 1
T1D: type 1 diabetes
TPR: tetratricopeptide repeat
TRIM_5αhu_
: human tripartite motif 5α
VSMC: vascular smooth muscle cell
ZF: zinc finger

## 1 Introduction

In 1984, Fisher and his colleagues investigated the presence of conformational transformation of proline containing peptides via enzymatic catalysis by examining homogenates from various biological material. They successfully purified and characterized an active protein from pig kidneys, which exhibited *cis–trans* peptidyl‐prolyl isomerase activity [1]. Concurrently, other research group purified a protein from bovine thymocytes that showed a high affinity for the immunosuppressive drug cyclosporine A (CsA) and named it cyclophilin [2]. Remarkably, 5 years later, it was discovered that both proteins were, in fact, the same entity, which was since recognized for both, its affinity to CsA and its pivotal enzymatic role, and termed as cyclophilin (Cyp) [3]. Later, other cyclophilin isoforms were identified, forming a whole new protein family, and the originally identified cyclophilin enzyme was assigned as cyclophilin A (CypA). In humans, 17 cyclophilins that share the typical structural feature, cyclophilin‐like domain (CLD), have been identified to date [4, 5, 6, 7]. They can be found in all cells of all organisms and they are present in all parts of the cell including cytosol, endoplasmic reticulum (ER), mitochondria, or nucleus and also extracellularly [8, 9, 10, 11, 12]. Today it is known that not all cyclophilins bind CsA, nor they all possess *cis*‐*trans* peptidyl‐prolyl isomerase (PPIase) activity [13, 14] (Table 1).

**Table 1:** List of human cyclophilins, their localization, aliases, PPIase activity, and CsA binding affinity.

Protein	Gene	Localization	Common aliases	PPIase activity	CsA binding
CypA	*ppia*	Cytosol, nucleus, extracellular	CypH, Cyp18	+	+
CypB	*ppib*	ER, nucleus, extracellular	Cyp22, Cyp‐S1	+	+
CypC	*ppic*	ER, cytosol		+	+
Cyp40	*ppid*	Nucleus, cytosol	cytosolic CypD	+	+
CypD	*ppif*	Mitochondrion, peroxisome, plasma membrane	Cyp3, CypF, mitochondrial CypD	+	+
CypE	*ppie*	Nucleus, cytosol	Cyp33	+	+
CypG	*ppig*	Nucleus, cytosol, extracellular	SRCyp, CARS‐Cyp, Cyp88, SCAF10	+	+
CypH	*ppih*	Nucleus, cytosol	Cyp20, USA‐Cyp, SnuCyp20, U4/U6‐20K	+	+
CypJ	*ppil3*	Nucleus, cytosol	CLK1, PPIL3	+	+
CypNK	*nk‐tr*	Nucleus, cytosol	NK‐TR, Cyp165	+	+
PPIL1	*ppil1*	Nucleus, cytosol	CypL1, hCypX	+	+
PPIL2	*ppil2*	Nucleus, golgi, cytosol	Cyp60, Cyp58, CYC4	−	−
PPIL4	*ppil4*	Nucleus, cytosol	Cyp57	−	−
PPIL6	*ppil6*	Cytosol, golgi	RSPH12, Cyp35	−	−
PPWD1	*ppwd1*	Nucleus, cytosol	Cyp73, Spliceosome‐associated Cyp, KIAA0073	+	+
RANBP2	*ranbp2*	Nucleus, cytosol	Cyp358, Nupp358	+	−
SDCCAG‐10	*cwc27*	Nucleus, cytosol	Cyp54, NY‐CO‐10	−	−

Cyclophilins are involved in protein folding, chaperone activity, regulation of immune function, pre‐mRNA splicing and many other processes. Due to their role in the broad range of physiological and also pathophysiological processes, cyclophilins represent a prospective class of drug targets. The main challenge for medicinal chemists presents their highly conserved active site, which interferes with development of the isoform specific inhibitors.

This review describes the general structure of cyclophilins and the specific structural features of particular cyclophilin isoforms along with their functions in health and disease. Furthermore, the current attempts and future possibilities for a development of selective cyclophilin inhibitors for a therapy of human diseases are discussed.

To our knowledge, there is no other review article covering all the 17 human cyclophilins, while comparing their structure, functions, and discussing the medicinal chemistry aspects of their inhibition. From the more recent literature we could mention reviews by Stauffer et al. [15] which covered three cyclophilins (CypA, CypB, and CypD), Rajiv and Davis [7], which covered the eight nuclear cyclophilins, or Bukrinsky [16], which covered the three extracellular cyclophilins. Other reviews usually focused on the role of cyclophilins in particular disease, for example, viral infections [17]. We would also like to acknowledge the seminal work in the field by Davis et al. [5].

### 1.1 General Structure of Cyclophilins

**Figure 1:**
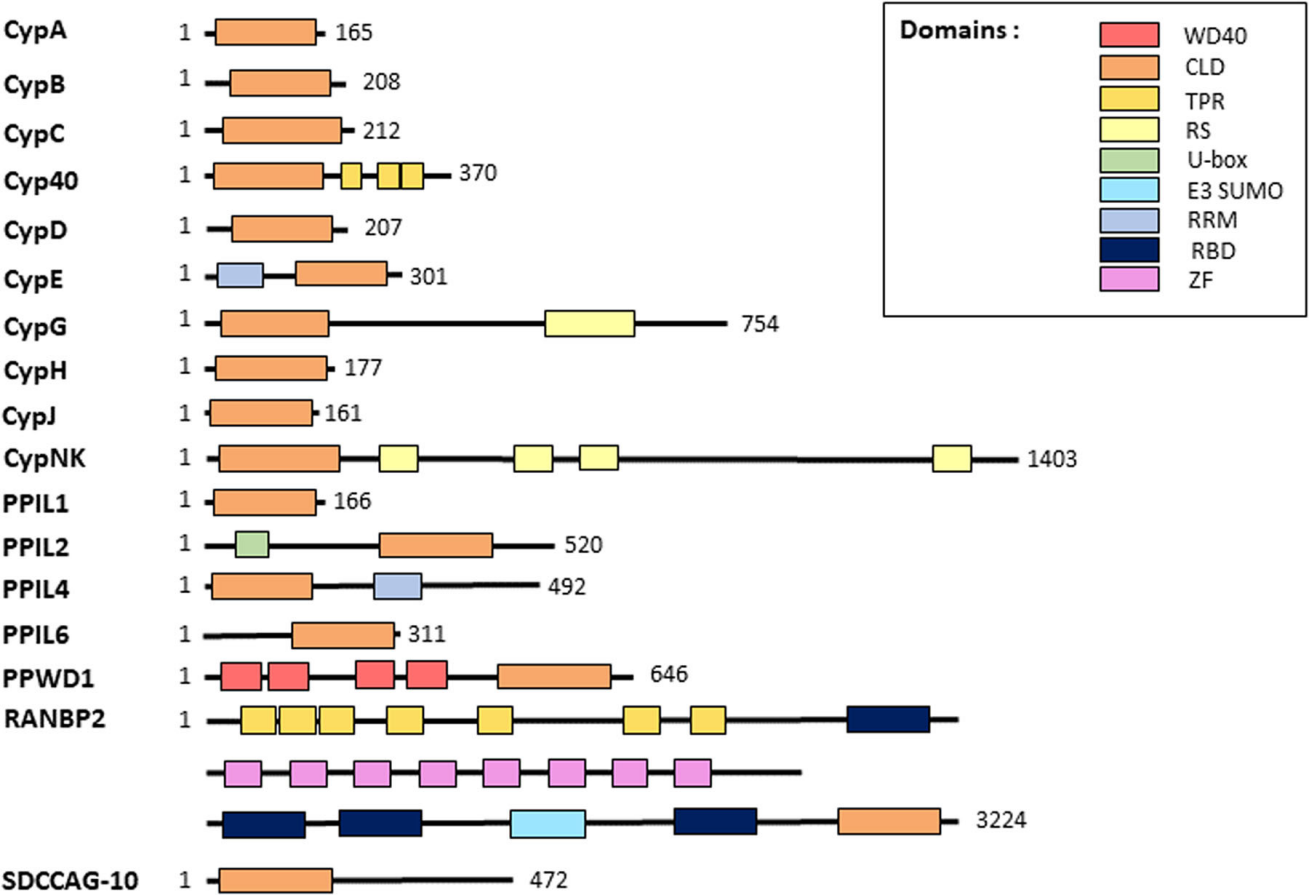
Domain organization of human cyclophilins. [Color figure can be viewed at wileyonlinelibrary.com]

A common structural feature of all cyclophilins is the possession of the CLD (also called the PPIase domain). The three‐dimensional structure of 14 individual CLDs was resolved so far. Structures of three remaining uncharacterized cyclophilins (Cyp40, PPIL4, and PPIL6) were predicted based on the data set of previously determined structures of other cyclophilins [5]. Generally, cyclophilins can be classified into two categories. First, single‐domain cyclophilins, which consist of a single CLD, and second multidomain cyclophilins, which have additional functional domains alongside the conserved CLD. Additional domains, for example, WD40, tetratricopeptide repeat (TPR), RNA recognition motif (RRM), or U‐box domain, are unique to each member of the cyclophilin family, and are associated with subcellular compartmentalization or functional specialization (Figure 1) [4, 18].

For illustration of the general structure of Cyps, we used the structure of CypA. We chose CypA because it was the first discovered Cyp. Its structure is well described, it consists only of CLD, and the later discovered isoforms were usually compared to it. Additional domains will be further discussed with the relevant isoforms. Residue numbering in this section corresponds to human CypA, unless otherwise stated.

**Figure 2:**
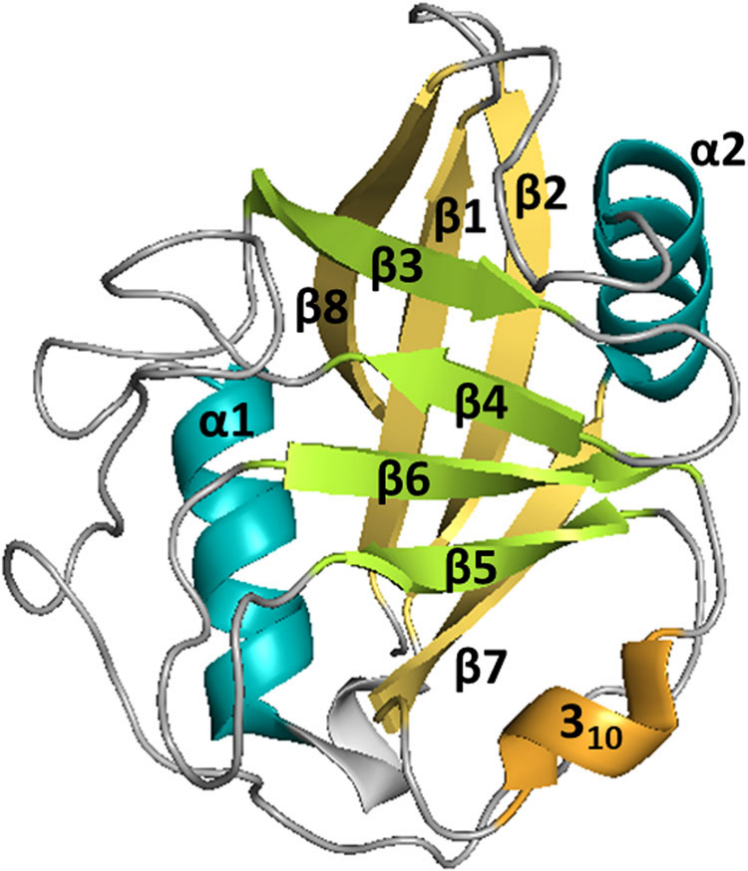
General structure of cyclophilins depicted on CypA structure (PDB ID: 2CPL). Characteristic structural components are color‐labeled. All Cyps contain the cyclophilin‐like domain, which consists of two β‐sheets (yellow and green), each one formed by four antiparallel β‐strands (β1, β2, β7, β8, and β3–6), two α‐helices (turquoise) and one short 3_10_‐helix (orange). Ribbon representation created using PyMOL software [19]. [Color figure can be viewed at wileyonlinelibrary.com]

Human CLD is composed of 139–197 amino acid (AA) residues and contains the highly conserved PPIase active site. CLD consists of two β‐sheets, each consisting of four antiparallel β‐strands, and two α‐helices that pack against the sheets. In the β6‐β7 loop region, there is one short α‐helical turn (also called 3_10_ helix) containing the important active site residue Trp121 that is mostly conserved among all Cyp isoforms (Figure 2) [5].

**Figure 3:**
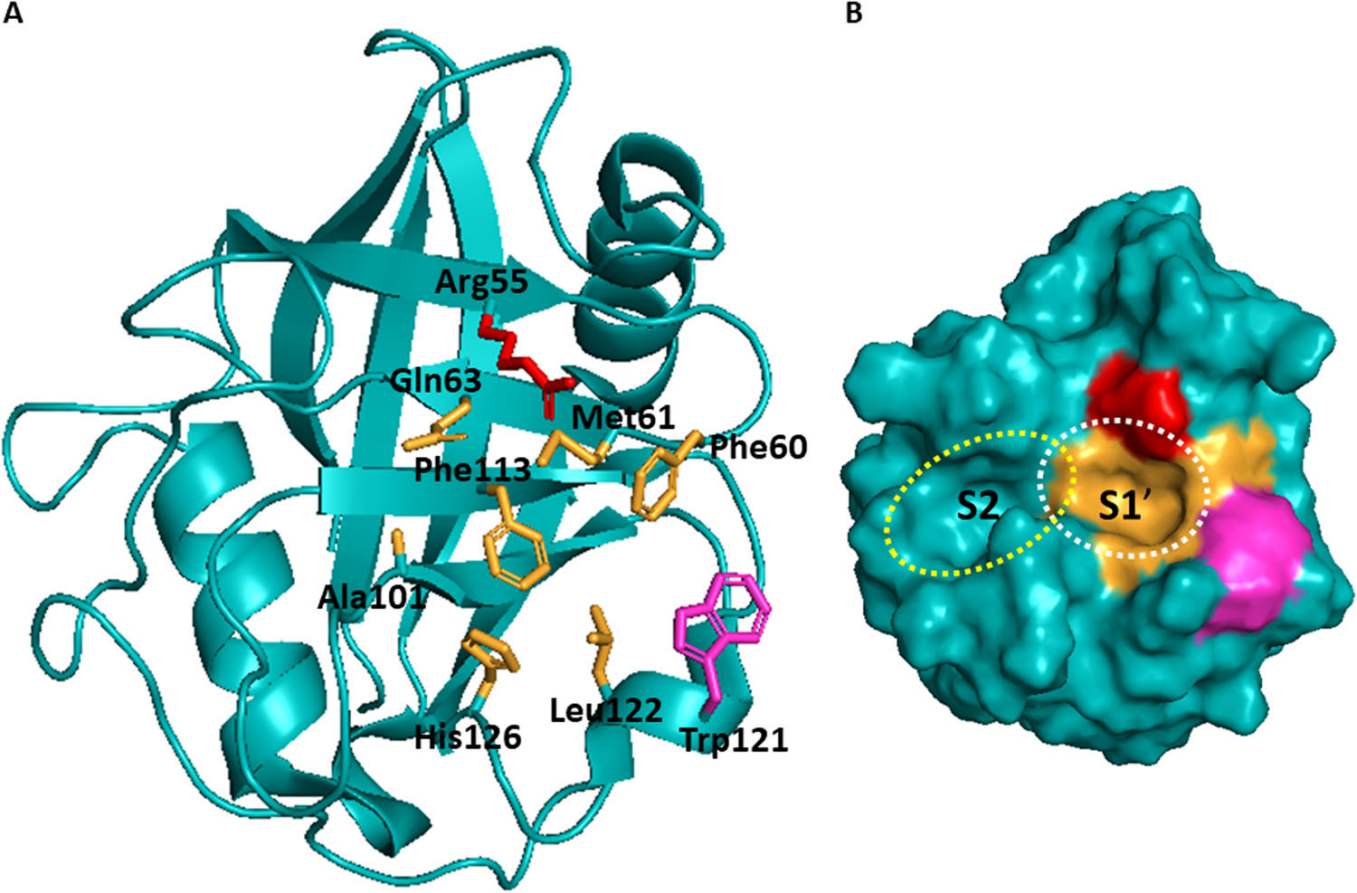
Active site of Cyps illustrated on CypA structure (PDB ID: 2PCL). (A) Cartoon representation of the active site with Arg55 highlighted in red, Trp121 highlighted in magenta, and the other important residues highlighted in orange. (B) Surface representation of the CypA and its active site. Binding pockets S1ʹ and S2 are highlighted with white and yellow dashed lines. Ribbon and surface representation created using PyMOL software [19]. [Color figure can be viewed at wileyonlinelibrary.com]

The active site contains the invariant arginine (Arg55) that is directly involved in the catalytic process. It is altered only in one isoform (PPIL4) that consequently lacks PPIase activity. Trp121 is other key residue within the active site in association with catalytic activity and also CsA binding. The experimental data showed that tryptophan is optimal at this position but histidine is also permissive for maintaining the enzymatic activity and CsA binding (except in RANBP2 which does not bind CsA). Four cyclophilins with Trp121 substituted for Tyr or Glu (PPIL2, PPIL4, PPIL6, and SDCCAG‐10) do not bind CsA and lack PPIase activity. There are several approaches of understanding the importance of Trp121 in active site. One suggests that the main role of Trp121 is to build a hydrophobic pocket for substrate proline [20, 21]. Another proposes that Trp121 is involved in specific polar interaction with the carbonyl moiety of methylleucine 9 (MLE9) in CsA or with the carbonyl of a substrate peptide at the P2’ position (where the sequence of the substrate AA_1_‐Pro‐AA_2_ is denoted P1, P1ʹ, and P2ʹ respectively). [5] Furthermore, the active site consists of a mixture of hydrophobic, aromatic, and polar residues including Phe60, Met61, Gln63, Ala101, Phe113, Leu122, and His126 (Figure 3), which can be altered in certain isoforms, but is generally highly conserved [22, 23, 24, 25].

To date, four binding pockets of cyclophilins were described, namely S1′ pocket (also called the catalytic, hydrophobic, proline, Mva, or Pro pocket), S2 pocket (also called the Aba, or Abu pocket), S1 pocket (also called the Bmt pocket), and three o'clock pocket (no alternative names). Two relatively uniform pockets across whole family of Cyps are S1ʹ and S2 pocket (Figure 3), which were firstly described by Davis et al. [5] as two adjacent binding pockets to the active site that contribute to substrate binding and turnover. S1ʹ pocket is proline binding site and the catalytic site for PPIase activity. Residues forming S1ʹ pocket are highly conserved and thus S1ʹ pocket will not contribute to the substrate specificity. The S2 pocket interacts with the second and third residue relative to the substrate proline. The base of the S2 pocket is defined by the main‐chain atoms of the β5‐β6 loop. The S2 pocket is uniform across the whole family of cyclophilins, thus it is relatively nonspecific. However, the set of “gatekeeper” residues on the sides of the pocket, which control access to the pocket, show significant chemical and size variance. Therefore, the gatekeeper residues of S2 pocket will be discussed later, together with S1 and three o'clock pockets, as potential sites for substrate/inhibitor selectivity (see Section 3.2 and Figure 23B,C).

### 1.2 Cyclophilins as Peptidyl‐Prolyl *cis–trans* Isomerases

**Figure 4:**
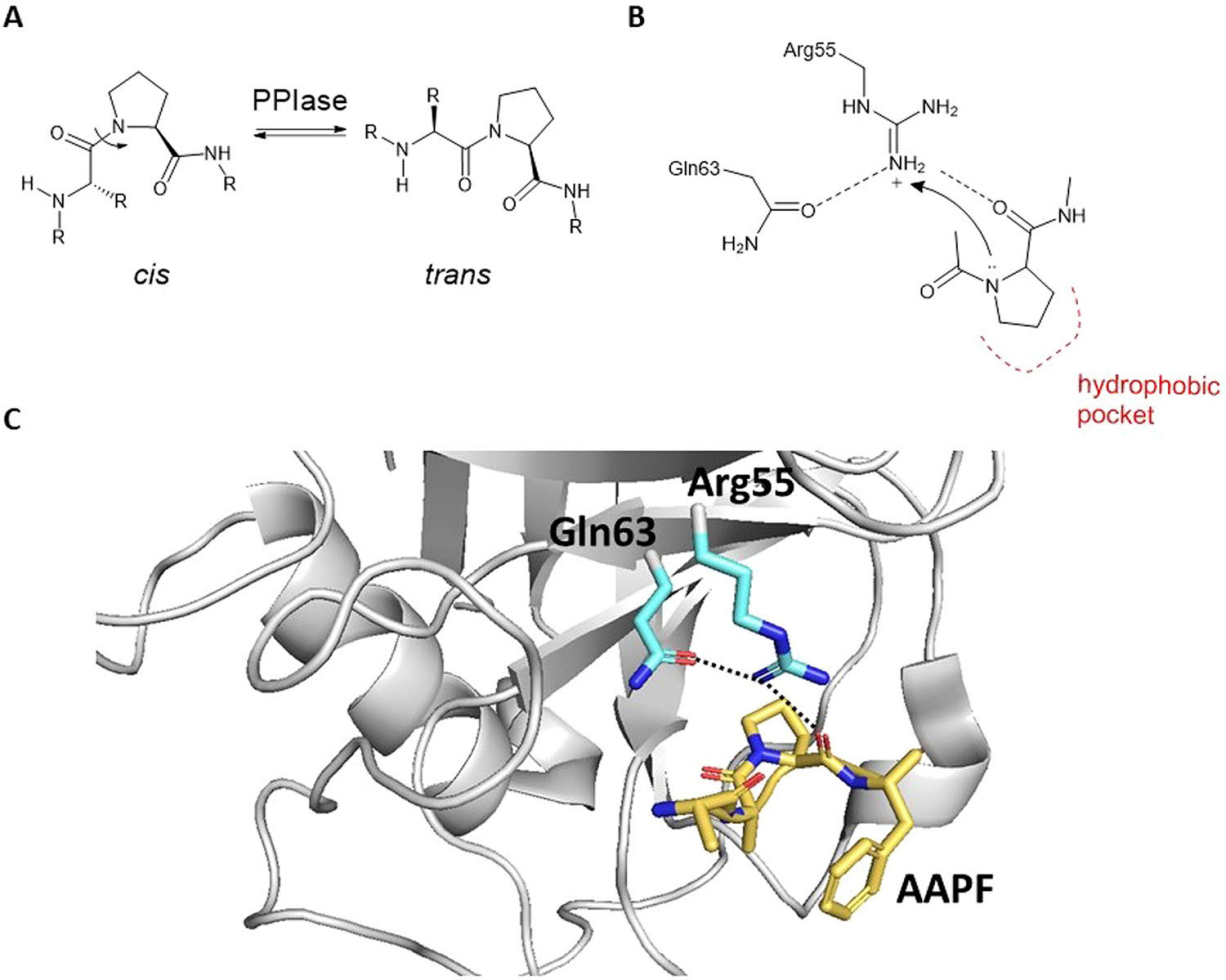
Peptidyl‐prolyl *cis–trans* isomerase activity of Cyps. (A) Illustration of *cis* and *trans* isomers of peptide bond to proline. (B) Schematic presentation of the catalytic mechanism by protonation on amide nitrogen with Arg55 as the catalytic group [26]. (C) Ribbon representation of crystal structure of CypA in complex with AAPF substrate (depicted in yellow). Black dotted lines represent hydrogen bonds (PDB ID: 2CPL). Ribbon representation created using PyMOL software [19]. [Color figure can be viewed at wileyonlinelibrary.com]

Cyclophilins possess *cis–trans* isomerase activity on the peptidyl‐prolyl amide bond (Figure 4A) [26, 27]. The amide bond has a partial double bond character and can exist either in *trans* or *cis* conformation. Ribosomes synthesize peptide bonds in a lower energy *trans* isomeric form. However, peptide bonds in proteins containing proline also exist in a *cis* isomeric form. Spontaneous isomerisation is a slow rate‐limiting step in protein folding that requires free energy. Cyclophilins accelerate isomerization by stabilizing the *cis–trans* transition state [28, 29].

On the basis of biochemical and structural studies, the two most likely mechanisms of isomerization have been proposed. First, “catalysis by distortion” mechanism, suggests that the Cyp binds and stabilizes a transition state of N‐C=O peptide plane bond that is distorted by partial rotation around the C‐N amide bond, while carbonyl group remains trigonal [30]. The second mechanism was proposed on the basis of quantum chemistry calculations and suggests that the sidechains of serine, threonine, or tyrosine protonate or form a hydrogen bond with the amide nitrogen to deconjugate the N‐C=O amide bond [31]. The mechanism is based on the fact that protonation on the amide nitrogen dramatically lowers the barrier to rotation between *cis* and *trans* forms (Figure 4B) [32]. The crystal structure of CypA in complex with AAPF substrate (succinyl‐Ala‐Ala‐Pro‐Phe‐*p*‐nitroanilide) was determined and supported this mechanism, although, as a main catalytic group was identified Arg55 instead of serine, threonine, or tyrosine (Figure 4C) [26, 33].

## 2 Cyclophilin Isoforms

Here, we provide an overview of the 17 known human cyclophilins. For each cyclophilin isoform, its structure, physiological functions, and the therapeutic potential of its pharmacological inhibition are discussed.

### 2.1 Cyclophilin A

Cyclophilin A (CypA) is the most abundant member of cyclophilin family (0.1%–0.6% of the total cytoplasmic proteins). It was first discovered in 1984 being the first known cyclophilin [34, 35]. It is a cytoplasmatic protein localized in all tissues of mammals and shares a high sequence similarity with CLD of other cyclophilin isoforms.

#### 2.1.1 Structure of CypA

**Figure 5:**
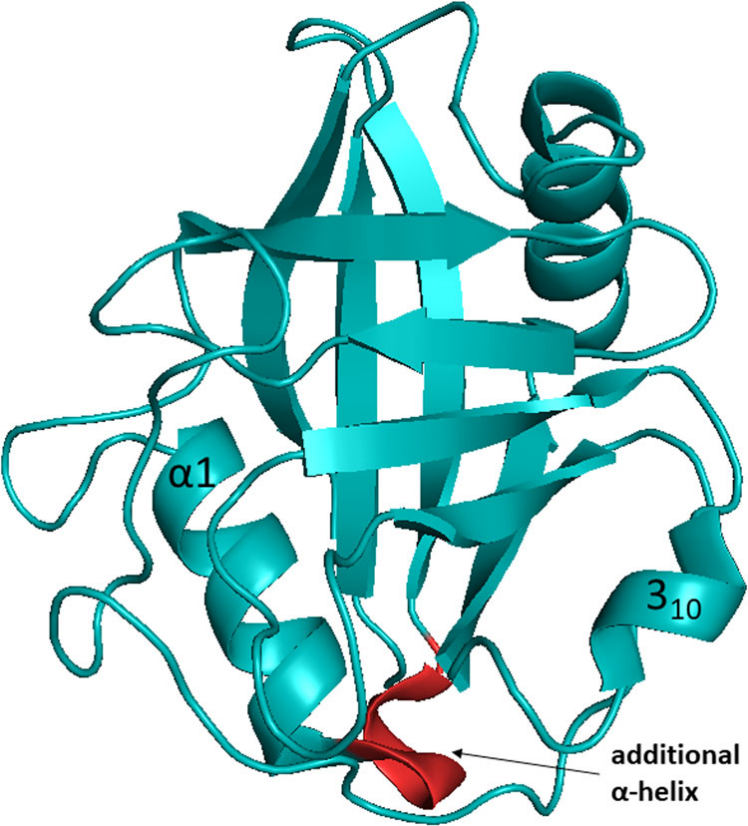
Crystal structure of CypA. The additional α‐helix is highlighted in red (PDB ID: 2CPL). Ribbon representation created using PyMOL software [19]. [Color figure can be viewed at wileyonlinelibrary.com]

CypA has a molecular mass of 18 kDa. In 1991, Ke et al. [36] resolved the three‐dimensional structure of unligated cyclophilin A (Figure 5). A single polypeptide chain with 165 AAs creates a secondary structure of two β‐sheets, each consisting of four antiparallel β‐strands, and two α‐helices covering the bottom and top of the barrel. Structure contains two short helices ‐ the typical short α‐helical turn (3_10_‐helix) in β6–β7 loop and an additional α‐helix formed by residues Phe25‐Lys28, which is followed by the common α1 helix (Figure 5). Such feature is present only in CypA and CypJ among the cyclophilin family. The active site for *cis*‐*trans* isomerization of a peptidyl‐prolyl amide bond is located on the barrel surface and contains residues Arg55, Phe60, Met61, Gln63, Phe113, Trp121, Leu122, and His126 [26].

#### 2.1.2 Function of CypA

CypA plays an important role in many biological processes, such as protein folding, intracellular trafficking, signal transduction, cholesterol metabolism, regulation of immune function, and inflammatory reaction of the body [11]. In addition, CypA participates in the pathophysiology of numerous conditions, including viral infections, cardiovascular, liver, and kidney disorders, neurodegeneration, cancer, as well as autoimmune diseases such as rheumatoid arthritis and psoriasis, diabetes, atherosclerosis, and aging [11, 35]. Due to the participation of CypA in many pathological conditions, it has become the most studied drug target among cyclophilin family members. Discussing the precise role of CypA in each mentioned disorder is beyond the scope of this article; for more information we refer to other reviews, such as those on CypA as a key player for human diseases [11] and CypA as a key player for infection of etiological agents [35]. Here, we focus only on those conditions where CypA inhibition represents a potential treatment strategy.

#### 2.1.3 CypA as a Drug Target

##### 2.1.3.1 Immunosuppression

Immunosuppressive effects of pan‐cyclophilin inhibitor CsA are mediated via its binding to CypA. Binary CsA/CypA complex interacts with calcineurin and inhibits its phosphatase activity. Ergo, CsA acts here as so called molecular glue enabling binding between the two proteins [37]. Inhibition of calcineurin results in prevention of events responsible for triggering immune responses, such as nuclear translocation of NF‐AT (nuclear factor of activated T‐cells), and IL‐2 (interleukin‐2) activation, resulting in immunosuppression [34]. CsA is used clinically, for example, in solid organ transplantation, rheumatoid arthritis, psoriasis, and amyotrophic lateral sclerosis (ALS). Notably, several nonimmunosuppressive CsA derivatives were prepared that still inhibit cyclophilins' PPIase activity, which indicate that CsA immunosuppressive effects are independent of the PPIase inhibition.

##### 2.1.3.2 Viral Infections

2.1.3.2.1

*Human immunodeficiency virus type 1*: The human immunodeficiency virus type 1 (HIV‐1) is the causative agent for the human acquired immunodeficiency syndrome (AIDS). After decades of research, AIDS remains mostly uncurable viral disease. However, there is a significant progress in understanding the pathogenesis of HIV. Interactions between Cyps and HIV‐1 capsid protein are considered to be the critical parts in life cycle of the HIV‐1. Despite the knowledge that CypA regulates the replication and cell‐entry of HIV‐1, by interaction with the residues 85–93 at CypA‐binding loop of the *N*‐terminal domain of HIV‐1 capsid [38], the precise mechanism of this interaction is still unknown. The most recent studies suggest that CypA acts either as a positive or negative regulator in HIV infection [39, 40, 41]. Positive regulation is accomplished by stabilizing the capsid, modifying the process of uncoating, improving the efficiency of reverse transcription and nuclear import. On the other hand, negative regulation is achieved by delaying capsid core uncoating and inhibiting the nuclear entry of HIV‐1 in a cell type‐dependent manner [35]. Selyutina et al. [42] studied the relationship of CypA and human tripartite motif 5α (TRIM5α_hu_) in HIV‐1 infection in lymphocytes. TRIM5α is a cellular restriction factor and a potent antiviral protein that restricts infection by HIV‐1 and other retroviruses [43]. Its antiviral activity is ascribed to induction of premature disassemble of the viral capsid. Both CypA and TRIM5α_hu_ bind to the HIV‐1 core, potentially competing for this interaction during infection. CypA appears to modulate the binding of TRIM5α_hu_ by sterically hindering its attachment to the core. This competition suggests that preventing CypA from binding allows TRIM5α_hu_ to effectively engage with the HIV‐1 core, thereby inhibiting infection. Based on these observations, inhibitors of CypA represent a promising therapeutic strategy for HIV‐1 treatment. Interestingly, in some primates has been identified a variant of the fusion protein TRIMCyp that combines the effector domain of the TRIM5α protein with the CypA capsid‐binding domain. CypA domain improves the binding specificity of TRIMα. The resulting protein represents an evolutionary advantage in antiviral defense against the HIV‐1 retrovirus [44].

*Hepatitis C virus*: Hepatitis C virus (HCV) belongs to the family of Flaviviridae viruses and causes hepatitis C, hepatic steatosis, cirrhosis, and hepatocellular carcinoma (HCC). The HCV genome encodes precursor polyprotein, which is cleaved into four structural proteins: core, E1, E2, and p7 and six nonstructural proteins: NS2, NS3, NS4A, NS4B, NS5A, and NS5B [45]. Many different studies confirmed importance of CypA in HCV replication [12, 46, 47, 48]. It is known that CypA interacts with nonstructural viral proteins NS5A, NS5B, and NS2 of HCV, and thus enhances the replication of HCV [49, 50, 51]. PPIase activity of CypA is crucial for interactions with these viral proteins, although the exact mechanisms are still unclear [52]. Several mechanisms by which CypA enhances HCV replication have been proposed, including recruiting NS5B into replicase [53], stimulating polyprotein proteolytic cleavage [54], promoting formation of membranous web [55], and increasing the RNA binding affinity of NS5A [56].

*Hepatitis B Virus*: Hepatitis B virus (HBV) is member of *Hepadnaviridae* family and is a causative agent of infectious hepatitis B, which affects liver and could lead to chronic hepatitis, cirrhosis, or HCC. CypA is involved in HBV replication, as in CypA‐silenced cells were levels of HBV DNA significantly reduced, as well as HBsAg (hepatitis B surface antigen) production and secretion from the cells. HBsAg affect pathogenesis during viral infection and is suggested to be an important factor in the impaired immune response [57, 58]. Moreover, Phillips and colleagues showed that CypA inhibition could interfere with intracellular formation and secretion of HBV viral and subviral particles. CypA is an important co‐factor for lipids and apolipoprotein B trafficking and cellular lipids are part of the HBV envelope proteins [59]. It should be also noted, that cyclophilin inhibitors (CsA and its nonimmunosuppressive analogs) exert an additional cyclophilin‐independent antiviral mechanism. They were found to inhibit HBV infection via blocking the viral entry by binding to the membrane transporter sodium taurocholate co‐transporting polypeptide (NTCP) with or without interfering with its transporter activity [60].

*Other viruses*: Among other representatives of flaviviruses belongs West Nile virus, Yellow fever virus, and Zika virus. Research showed that CypA interacts with a nonstructural protein NS4B of flavivirus, and thus regulates viral replication. Vidotto et al. [61] also found that CypA inhibition efficiently reduces viral infection. The mechanism of CypA‐NS4B interaction is not fully understood yet, however, NS4B could be a possible target for flavivirus therapy.

Another group of viruses affected by CypA is *Nidovirales*. Nidoviruses include human viruses such as the MERS and SARS‐coronavirus (SARS‐CoV) and some other animal viruses [62]. Coronaviruses are known to cause serious respiratory diseases such as severe acute respiratory syndrome (SARS) or, more recently discovered, coronavirus disease 2019 (COVID‐19) that caused a global pandemic in 2020. Previous studies showed that CypA interacts with a nonstructural protein Nsp1 of the *N*‐terminal part of SARS‐CoV and gets incorporated into SARS‐CoV particles [63]. Several studies showed that CypA interacts with the coronavirus nucleocapsid protein, which plays crucial role in host cell entry, as well as in virus particle assembly and release [64, 65, 66]. Study by Ma‐Lauer et al. [66] have also shown that Cyp inhibitors disrupt this interaction, highlighting their potential to suppress viral replication. Later, study in 2011 by Pfefferle et al. [67] and in 2014 by Carbajo‐Lozoya et al. [68] confirmed involvement of CypA in replication of coronaviruses by showing that cyclophilin inhibitor CsA completely inhibited virus replication. Furthermore, in 2020 Softic et al. [69] showed that a non‐immunosuppressive analog of CsA, alisporivir, inhibits the infection of SARS‐CoV‐2 by inhibiting a postentry step of the SARS‐CoV‐2 life cycle. Recent study in 2023 by Sheng et al. [70] showed that CypA stabilizes SARS‐CoV‐2 spike protein by facilitating spike folding and trimer formation resulting in increased viral infectivity. Use of Cyp inhibitors prevents infection and emphasizes CypA as a target for antiviral therapy. However, it should be noted that role of CypA in human coronavirus infections (resp. replication) is not uniform. CypA involvement depends on the virus subtype, infected cell type, and the specific stage of the viral life cycle. For instance, CypA is required for HCoV‐NL63 infection but not for HCoV‐229E or MERS‐CoV infection [71].

Taken together, CypA plays an important role in many different viral infections but there is only a little known about the exact mechanisms of action, which need to be further elucidated to gain the future perspective in searching for the potential antiviral drugs.

##### 2.1.3.3 Cardiovascular Diseases

In 2000, Jin et al. [72] identified CypA as a secreted oxidative stress‐induced factor. They discovered that CypA acts as a secreted redox‐sensitive mediator that stimulates extracellular signal‐regulated kinase 1/2 (ERK1/2) activity, promotes vascular smooth muscle cell (VSMC) proliferation, inhibits VSMC apoptosis, and exhibits increased expression and secretion in the presence of sustained intracellular reactive oxygen species (ROS) generation and after vascular injury. Inflammation triggered by oxidative stress is the cause of many human cardiac diseases such as abdominal aortic aneurysm (AAA), atherosclerosis, hypertrophy, ischemia‐reperfusion injury (IRI), and coronary artery disease [11, 73].

*Abdominal Aortic Aneurysm*: The pathophysiology of AAA is related to an initial arterial insult causing cascade of inflammation and extracellular matrix protein breakdown by proteinases leading to arterial wall weakening [74]. CypA as a factor influencing mechanisms appearing in formation of AAA is considered to be a promising target for treating the disease [72, 75, 76, 77]. In 2009, Satoh et al. [75] found that CypA is essential mediator of AAA formation and characterized four pathological mechanism of AAA formation promoted by vascular CypA. First, secretion of CypA is promoted by angiotensin II (AngII)‐induced ROS. Second, secreted extracellular CypA contributes to the production of ROS synergistically with AngII in VSMCs. Third, CypA promotes matrix metalloproteinase‐2 (MMP‐2) activation by inducing membrane type‐1 MMP (MT1‐MMP) activation and increasing the formation of ROS. Finally, recruitment of CD45^+^ inflammatory cells is stimulated by CypA. In 2012, Prins et al. [78] discovered that benzo[*a*]pyrene increases CypA expression and thus potentiates AAA formation. At the same time, the next study showed the effectivity of simvastatin in the inhibition of CypA expression in 2013 [79]. Treatment with simvastatin decreased CypA mRNA and CypA intracellular protein levels, thus statins were proposed a new treatment strategy for patients with AAA.

*Atherosclerosis*: Atherosclerosis is disease characterized by inflammation, lipid accumulation, cell death and fibrosis. Depending on the location, it can lead to serious conditions such as myocardial infarction, heart failure, ischemic stroke, renal failure, hypertension, and AAA [80]. In 2010, Nigro et al. [81] found that CypA deficiency in vivo decreases atherosclerotic lesion burden in a mouse model and characterized five pathological mechanisms, by which CypA promotes atherosclerosis. First, CypA regulated the scavenger receptor expression, and thus increases low density lipoprotein uptake in the vessel wall. Second, CypA enhanced endothelial cell activation and inflammation through increased expression of the vascular cell adhesion molecule 1. Further, CypA decreased endothelial nitric oxide synthase expression by transcriptional repression of Kruppel‐like factor 2. And, CypA was a key determinant of TNF‐induced endothelial cell apoptosis. Finally, CypA stimulated the recruitment of inflammatory cells derived from bone marrow to the aortic wall. Another pathological mechanism involves extracellular CypA. Multiple studies have shown that activation of the CD147 receptor by extracellular CypA initiates a cascade of pro‐inflamatory processes that contribute to vascular inflammation and plaque instability (more details in Section 2.1.3.7) [82, 83, 84].

##### 2.1.3.4 Neurodegeneration

*Alzheimer's disease (AD)*: AD is the most common cause of dementia in the elderly. The exact mechanisms are not yet fully understood, but are believed to involve pathological mechanisms such as extracellular amyloid‐β (Aβ) deposition [85], tau protein aggregation [86], oxidative stress [87], mitochondrial dysfunction [88, 89], and a decrease in acetylcholine levels [90]. Many studies showed an association of CypA with oxidative stress, which is considered to be involved in different neurodegenerative diseases including AD [11, 72, 91]. In 2012, Bell et al. [92] showed that CypA initiates a pro‐inflammatory pathway activating nuclear factor kappa B (NF‐κB) and matrix metalloproteinase‐9 (MMP‐9), which leads to an age‐dependent progressive blood‐brain barrier (BBB) breakdown driven by astrocyte‐derived human ApoE4 (apoliprotein E4). This leads to the release of blood‐derived neurotoxic molecules (e.g., fibrinogen, thrombin, plasminogen, erythrocyte‐derived free iron, and antibrain antibodies) that damage neurons and affect their synaptic connections [11, 93].

*ALS*: ALS is a neurodegenerative disease that implicates central and peripheral motor neurons [94]. Familial ALS (10% of all cases) is believed to be caused by mutations in the superoxide dismutase 1 gene (SOD1) [95]. Protein aggregation was considered to play an important role in the pathogenesis of familial ALS [96]. Increased concentrations of CypA were found in the Triton X‐100 insoluble fraction (TIF) of ALS spinal cord, which may indicate a relationship of CypA with protein aggregation [11, 97]. Pasetto et al. [98] found that extracellular CypA is a mediator of neuroinflammation in ALS having toxic effects on motor neurons. Selective inhibition of extracellular CypA can lead to a reduction in neuroinflammation and protection of neurons. In another study, a CsA derivative MM218 was tested for its inhibitory effect and confirmed the hypothesis of motor neuron protection [99, 100].

##### 2.1.3.5 Cancer

CypA is involved in development of various types of cancer. Overexpression of CypA has been observed in liver [101, 102, 103], pancreatic [104, 105, 106], lung [107], esophageal [108], endometrial [108, 109, 110], breast [111, 112], gastric [113], and melanoma cancers [114]. CypA takes part in tumor proliferation, invasion, and metastasis through deregulation of its isomerase activity [115]. It is also associated with acquired chemoresistance [116, 117]. However, the exact mechanisms of CypA action in cancer are still poorly understood [118].

##### 2.1.3.6 Nonalcoholic Steatohepatitis (NASH)

NASH is a chronic liver disease that could progress even to liver cancer. Three cyclophilin isoforms have been shown to play a role in disease pathophysiology, namely CypA, CypB, and CypD. NASH is accompanied with increased oxidative stress, which elevates extracellular levels of CypA. Interaction of CypA with pro‐inflammatory receptor CD147, promoted infiltration and activation of inflammatory cells and resulted in promotion of fibrosis [119].

##### 2.1.3.7 Inflammatory Diseases

It is important to mention that CypA (together with CypB and CypC) can also be found extracellularly, where it acts as a pro‐inflammatory factor implicated in pathogenesis of a number of inflammatory diseases [16]. A critical feature of extracellular CypA is its interaction with the signaling receptor CD147 (also known as EMMPRIN), which drives chemotaxis and promotes inflammatory responses [100, 120, 121, 122]. Activation of CD147 in leukocytes has been implicated in processes underlying lung injury, rheumatoid arthritis, chronic liver disease, heart failure, artherosclerosis, and biliary atresia and thus inhibition of extracellular CypA represents a promising candidate for intervention in such conditions [82, 123].

### 2.2 Cyclophilin B

Cyclophilin B, also known as Cyp22 or Cyp‐S1, is a single‐domain protein located in the endoplasmic reticulum. CypB was firstly isolated in 1991, by Price et al. [124] and was found in the most tissues with the highest expression levels in thyroid gland, testis, colon, and skin.

#### 2.2.1 Structure of CypB

**Figure 6:**
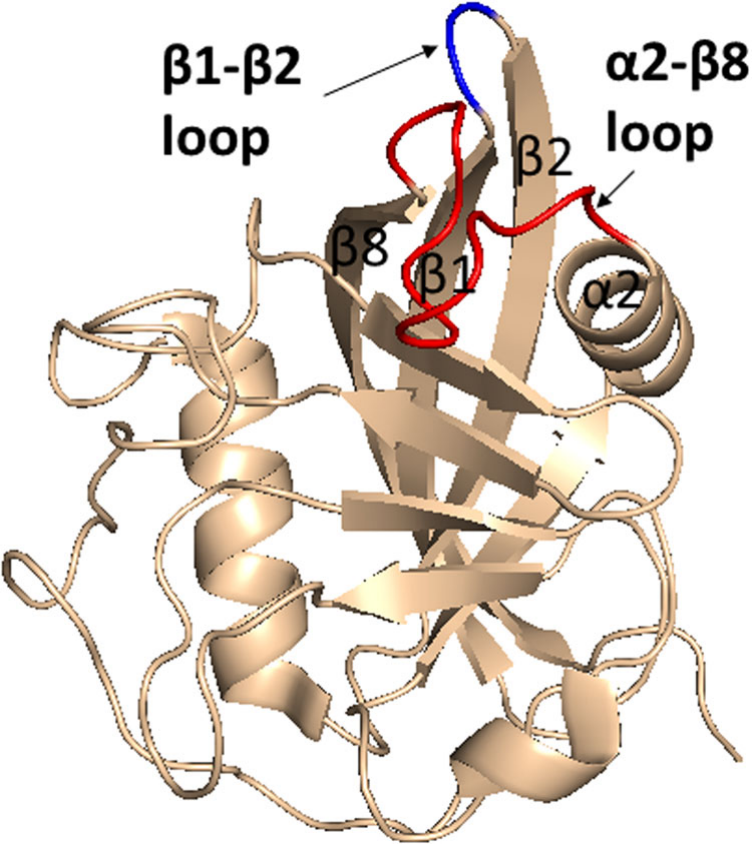
Structure of CypB. Crystal structure of CypB includes residues 7–184. Diverse β1‐β2 and α2‐β8 loops in comparison to CypA are highlighted in blue and red (PDB ID: 1CYN). Ribbon representation created using PyMOL software [19]. [Color figure can be viewed at wileyonlinelibrary.com]

CypB, a 22 kDa protein, contains 208 AAs and shares 64% sequence identity to CypA [124]. In comparison to CypA, CypB contains additional 33 AAs long signaling sequence at the *N*‐terminus, which is considered to direct CypB to the endoplasmic reticulum. Additional 10 AAs can be also found at *C*‐terminus. Remaining 165 AAs make up the CLD of CypB [124]. In 1994, Mikol et al. [125] characterized X‐ray structure of CypB/CsA complex. They showed that CypB differs from CypA in the folding of two loops (residues 19–24 of β1‐β2 loop and residues 152–164 of α2‐β8 loop, Figure 6) and in the extensions at the *N*‐ and *C*‐termini. The binding pocket of CypB shows no significant differences from CypA. However, the inhibition of PPIase activity by CsA shows different values for CypA (IC_50_ = 25 nM) and CypB (IC_50_ = 84 nM) [124]. In addition, CypB/CsA complex inhibits calcineurin 13‐fold more (*K*_*i*_ < 21 nM) compared to CypA/CsA complex (*K*_*i*_ = 336 nM) [126].

#### 2.2.2 Function of CypB

Despite the high degree of similarity between CypB and CypA, their functions differ. CypB acts as a regulator of collagen folding [127] and as an intracellular chaperone for calcium‐modulator (CAML) [128], interferon regulatory factor 3 (IRF‐3) [129], and prolactin [130]. CypB binds to Gag protein of HIV‐1 and also plays a role in other viral infections such as HCV [131], Japanese encephalitis virus (JEV) [132], and human papillomavirus type 16 (HPV16) [133].

CypB binds to CsA, forming the CypB/CsA complex, which in vitro inhibits calcineurin even more potently than the CypA/CsA complex. However, in vivo CypB is likely not the relevant immunosuppressant binding protein in T‐cells since it resides in the ER [126].

In 2016, CypB was found to have positive effect in Parkinson's disease (PD), a neurodegenerative disease characterized by progressive loss of dopaminergic neurons in the substantia nigra, striatum, and putamen [134]. Oh et al. [135] conducted a study on involvement of CypB in neuronal cell death induced by neurotoxin MPP+ (model of PD). They confirmed that overexpression of CypB protects SH‐SY5Y human neuroblastoma cells from apoptosis by inhibition of JNK activation.

A study of osteogenesis imperfecta conducted by Choi et al. [136] showed that CypB plays a critical role in facilitating proper collagen formation and bone formation. CypB most likely promotes the proliferation and differentiation of MC3T3‐E1 cells via the JAK2/STAT3 signaling pathway [137].

#### 2.2.3 CypB as a Drug Target

##### 2.2.3.1 Viral Infections

*HIV‐1*: In 2015, DeBoer et al. [138] examined the role of CypB in HIV‐1 infection. They concluded that overexpressed intracellular CypB promotes HIV‐1 infection through increased nuclear import of HIV DNA. They suggested two possible mechanisms of action. CypB interacts either with the viral capsid and activates cellular pathways or promotes infection by interaction in the perinuclear region. Clarification of the exact mechanism could provide a new treatment strategy.

*HCV*: In 2005, Watashi et al. [131] studied HCV genome replication and attempted to identify involved cellular factors by using CsA as it was previously shown to suppress HCV genome replication [46, 139, 140]. They identified CypB as a factor necessary for HCV genome replication. CypB interacts with viral protein NS5B and thus modulates its RNA binding activity. As a positive regulator of HCV replication, CypB presents an interesting therapeutic target.

*Orf virus (ORFV)*: ORFV, a member of *Poxviridae*, causes a contagious skin disease in sheep and goats, also known as contagious ecthyma or sore mouth [141]. The disease can be transmitted to humans through direct contact with an infected animal [142]. To investigate the role of CypB in the replication of ORFV, Zhao et al. [142] conducted a study using ORFV‐infected MDBK (Madin‐Darby bovine kidney) cells. Upregulation of CypB was found in ORFV‐infected MDBK cells. In addition, use of CsA suppressed ORFV replication and silencing of CypB gene inhibited the replication of ORFV.

*JEV*: The JEV is one of the most important flaviviruses spread mainly in Eastern and Southeast Asia. The virus is transmitted by mosquito bites among pigs, birds, and also humans. In 2011, Kambara et al. [132] found that CypB interacts with viral protein NS4A indicating its important role in the replication of JEV.

##### 2.2.3.2 AD

One of the pathological mechanisms occurring in AD is the accumulation of extracellular Aβ. The precise mechanism is still not known, but data show that Aβ contributes to synaptic dysfunction, disruption of neuronal connectivity, and neuronal death [143]. Neuronal cell death may also be caused by Aβ disrupting Ca^2+^ homeostasis or triggering oxidative stress in ER or mitochondria [144, 145, 146, 147]. In 2008, Kim et al. [148] showed that CypB is an important ER stress regulator. Overexpression of CypB protects cells from ER stress. Based on this knowledge, Oh et al. [147] conducted a study on the neuroprotectivity of CypB. They found that CypB reduced oxidative stress induced by Aβ and prolonged the life span of neurons through signaling pathways of mitogen‐activated protein kinase (MAPK) and phosphoinositide 3‐kinase (PI3K). The fact that overexpression of CypB was found to have neuroprotective, antioxidative, and antiapoptotic effects points to the importance of development of selective cyclophilin inhibitors, since CypA, CypD, and PPIL2 have opposite (pro‐AD) effect and their inhibition is considered to be applicable for AD treatment.

##### 2.2.3.3 Cancer

Overexpression of CypB was observed in breast, liver, colon, pancreatic, and stomach cancer, and CypB was found to play a role in the malignant progression of tumors [149, 150, 151, 152]. Fang et al. [149] demonstrated that CypB enhanced the effects of prolactin (PRL) in the pathogenesis of breast cancer. They suggested that CypB regulates PRL‐responsive genes through the activation of receptor expression (i.e., PRLR), chaperoning of the ligand (i.e., PRL), and inducing the transcriptional factor (i.e., Stat5). Kim et al. [150] showed that overexpression of CypB leads to the promotion of cancer cell viability in response to oxidative stress. The secretion of overexpressed CypB and its binding to CD147 protected hepatoma cells against apoptosis through the ERK activation pathway. Downregulation of CypB resulted in inhibition of proliferation, migration, and invasion [153, 154]. Lee et al. [154] studied the inhibition mechanism of Honokiol (HNK) in cancer cell migration. They suggested that HNK acts by targeting the CypB signaling pathway. HNK lowered CypB expression, which resulted in suppression of cell migration.

##### 2.2.3.4 Nonalcoholic Steatohepatitis

CypB was (together with CypA and CypD) associated with the development of nonalcoholic steatohepatitis (NASH). Elevated serum levels of CypB were found to be pro‐inflammatory. In addition to its pro‐inflammatory effects, CypB also participates in the promotion of fibrosis via interaction with CD147. Thus, CypB inhibition presents a promising therapeutic strategy for NASH [119].

### 2.3 Cyclophilin C

CypC is other member of the cyclophilin family (alongside CypB) localized to the ER. Human CypC was firstly isolated and described in 1994. It was found to be expressed in kidney, pancreas, skeletal muscle, heart, lung, and liver [155].

#### 2.3.1 Structure of CypC

**Figure 7:**
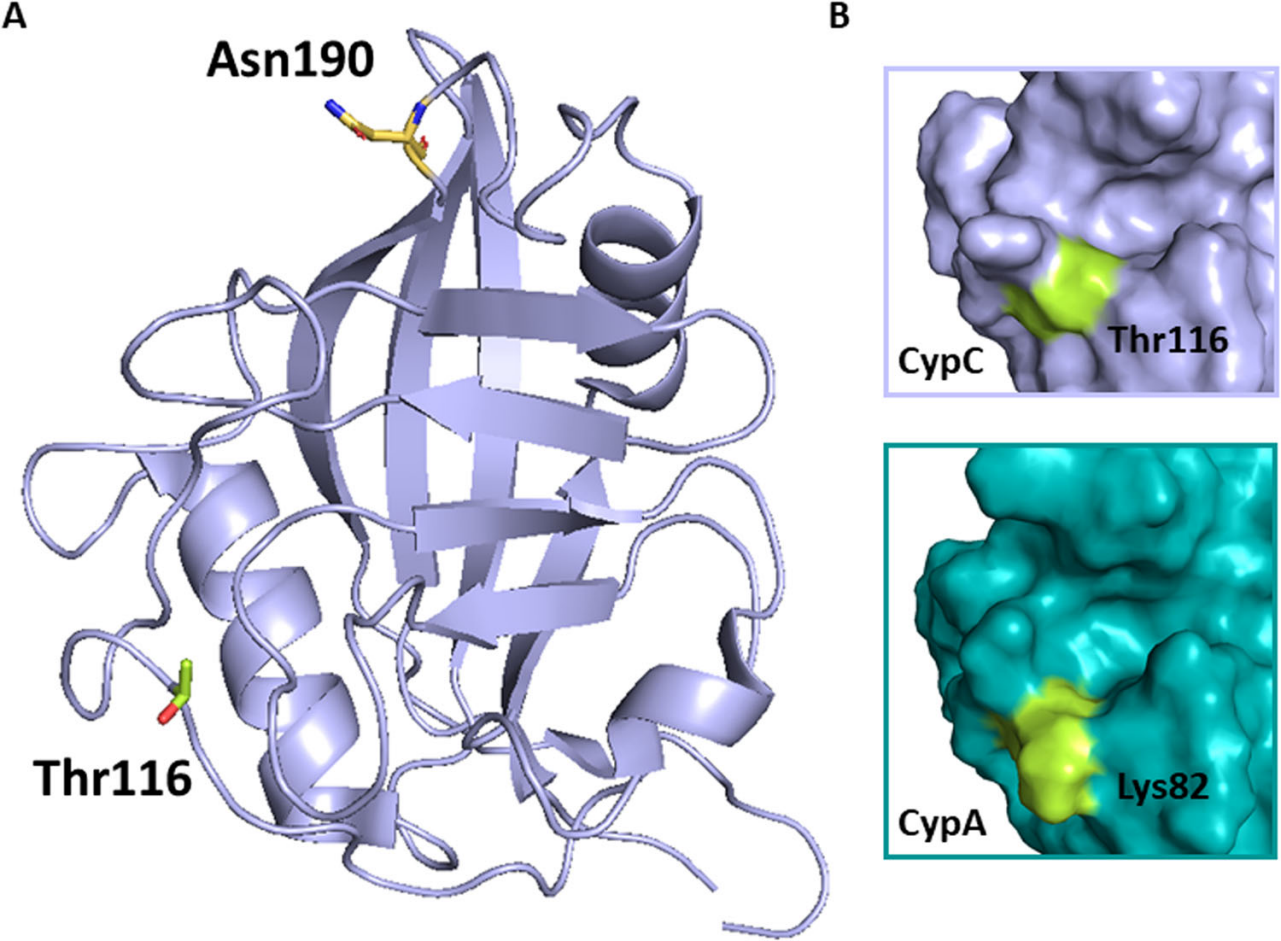
Structure of human CypC. (A) Crystal structure of human CypC with unique Thr116 highlighted in green and Asn190 highlighted in yellow (PDB ID: 2ESL). (B) Surface representation of Thr116 in human CypC which corelates with Lys82 in CypA. Threonine in this position generates more space for binding to S2 pocket (PDB IDs: 2ESL, 2CPL). Ribbon and surface representation created using PyMOL software [19]. [Color figure can be viewed at wileyonlinelibrary.com]

CypC shares the highest sequence identity (67%) with CypB. The high structural similarity is partially due to the same ER signaling region. Crystal structure of human CypC (Figure 7A) was resolved by Davis et al. [5] Human CypC consists of 212 AAs where 190 AAs form typical CLD and rest are the ER signaling sequence and membrane interacting residues. The *N*‐ and *C*‐terminal residues extend to the outside of the β‐barrel and may interact with the cell membrane or other cellular organelles [156]. The *N*‐terminus bears a hydrophobic ER signaling sequence [156, 157]. In the position where the second residue of CsA binds to the S2 pocket of cyclophilin, there is a Lys82 (gatekeeper 2) of CypA replaced by a Thr116 in CypC which generates more space (Figure 7B) in the S2 pocket, resulting in higher tolerance for modifications of CsA in position 2 [155].

In 2014, Stocki et al. [158] mentioned two species of CypC: an endoglycosidase H‐sensitive‐glycosylated form and unglycosylated form. The surface‐located and unique CypC residue Asn190 is most likely to be the *N*‐glycosylation site.

#### 2.3.2 Function of CypC

Although the main physiological role of CypC is still uncertain, CypC was found to modulate macrophage activation, endotoxin signaling, and metalloproteinkinase‐13 expression via binding to the CypC‐associated protein (CypCAP) [156, 158, 159, 160]. CsA inhibits the binding of CypCAP to CypC, suggesting that they share the same binding site.

CypC was associated with coronary artery disease (CAD) in addition to several other cyclophilins (CypA, CypB, and CypD) [161, 162, 163, 164]. Further, CypC was found to have a neuroprotective effect in association with cerebral ischemia. Shimizu et al. [165] investigated the role of CypC and CypCAP in cerebral infarction and they proposed that CypCAP acts as endogenous CypC ligand with neuroprotective effect.

#### 2.3.3 CypC as a Drug Target

##### 2.3.3.1 Cytomegalovirus

Cytomegalovirus is a member of *Herpesviridae*. Human cytomegalovirus is recognized by major histocompatibility complex class I molecules (MHC class or MHC I). The viral mechanism to avoid immune recognition by MHC class I is mediated by the immunoevasin protein US2, which plays a role in ER‐associated degradation pathway resulting in destruction of newly synthesized class I molecules [166]. In 2015, Chapman et al. [166] identified CypC as a component of US2‐mediated degradation of MHC class I. They found that CypC expression needs to be at particular level because both, the depletion as well as its overexpression, impaired US2‐mediated avoid mechanism.

### 2.4 Cyclophilin 40 (Cytosolic CypD)

To begin with, it should be noted that the CypD designation can be used for two different proteins, namely cytosolic CypD and mitochondrial CypD. Cytosolic CypD is encoded by the peptidyl‐prolyl *cis*‐*trans* isomerase D gene (*ppid*), and mitochondrial CypD is encoded by the peptidyl‐prolyl *cis*‐*trans* isomerase F gene (*ppif*) gene. In this review, cytosolic CypD will be referred to as Cyp40 and mitochondrial CypD simply as CypD.

#### 2.4.1 Structure of Cyp40

**Figure 8:**
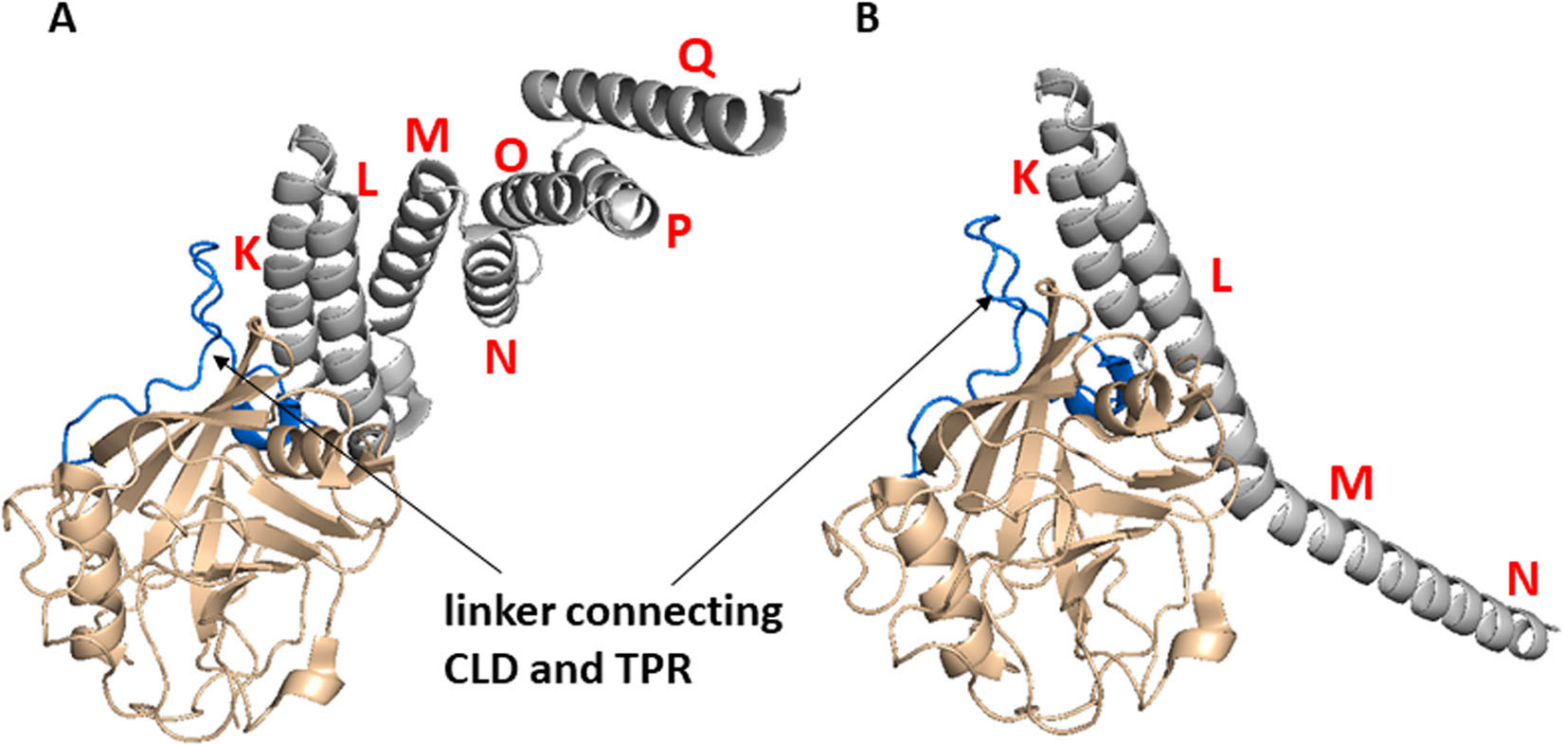
Structures of bovine Cyp40. The cyclophilin‐like domain is depicted in beige. Linker between CLD and TPR domain is depicted in blue. TPR domain is depicted in gray. (A) Crystal structure of the monoclinic form of bovine Cyp40 (PDB ID: 1IHG). (B) Crystal structure of the tetragonal form of bovine Cyp40. Three helices (O, P, and Q) are not visible (PDB ID: 1IIP). Ribbon representation created using PyMOL software [19]. [Color figure can be viewed at wileyonlinelibrary.com]

Cyp40 is a multidomain Cyp that has an additional *C*‐terminal tetratricopeptide repeat (TPR) domain. The whole sequence of Cyp40 consists of 370 AAs with molecular weight of 40 kDa. Crystal structure of human Cyp40 was not determined to date. However, bovine Cyp40 is highly homologous to human version with only three AA residues substituted, and thus here we provide the example of bovine Cyp40 (Figure 8A,B). Moreover, predicted structure of human Cyp40 does not differ from bovine Cyp40. Two structures of bovine Cyp40 were described: monoclinic and tetragonal (Figure 8) [167]. CLD in both structures of Cyp40 contains 183 AA residues and features the typical CLD fold. The active site of Cyp40 is identical to CypA, except for His141 instead of Trp121 in CypA. This change, however, does not hamper its PPIase activity or its affinity to CsA. The 30 AAs long linker between CLD and TPR domain forms 2 β‐turns and contains 11 Asp and Glu residues, making it more acidic. Conformation of TPR domain differs in monoclinic and tetragonal form [167]. In monoclinic form, TPR domain consists of seven helices (K–Q; each TPR motif contains 34 AAs). In tetragonal form, two of the helices are straightened out to form one extended helix (Figure 8B).

#### 2.4.2 Function of Cyp40

The biological role of Cyp40 is still not fully understood, but it is known that large immunophilins such as FKBP52, FKBP54, or Cyp40, which contain the TRP domain, are binding to the heat shock protein 90 (Hsp90) to regulate steroid receptor activity [168]. The residues important for Hsp90 binding are located in the TPR domain. Inhibition of Cyp40 interaction with Hsp90 could present a potential target for a cancer or inflammation therapy. On the other hand, Cyp40 and Hsp90 binding has a cytoprotective effects during IRI [169]. It was suggested that CLD of Cyp40 plays a role in identifying partner proteins after phosphorylation and in signaling a response to oxidative stress [167].

#### 2.4.3 Cyp40 as a Drug Target

##### 2.4.3.1 Hepatitis C Virus

Cyp40 was found to play an important role in HCV replication, besides CypA and CypB [170]. As a molecular chaperone, Cyp40 interacts with the viral proteins and assists their function. Given the fact that Cyp40 binds to Hsp90, it is assumed that it acts as a linker between viral proteins and Hsp90. Thus, Cyp40 presents a potential antiviral target [170].

##### 2.4.3.2 Prostate Cancer (PCa)

PCa is the most commonly diagnosed cancer in males worldwide [171]. The growth of PCa is dependent on androgens and the androgen receptor (AR). Cyp40 is known to interact with AR in PCa cells affecting their transcription and cell growth. In 2010, Periyasamy et al. [172] reported that Cyp40 levels in PCa tissues are elevated and that Cyp40 is a positive regulator of AR. Therefore, the inhibition of Cyp40 provides a potential strategy in PCa treatment.

### 2.5 Cyclophilin D (Mitochondrial)

Mitochondrial cyclophilin D (CypD) was discovered in 1990, when it was isolated from rat liver and heart [173]. CypD is localized in the mitochondrial matrix and is expressed in all human tissues with the highest expression rates in liver, heart, and kidney [119, 174, 175].

#### 2.5.1 Structure of CypD

CypD is a single‐domain protein containing 207 AAs with total molecular weight of 22 kDa. It contains CLD consisting of 165 AAs and a mitochondrial targeting sequence [5]. Mitochondrial targeting sequence includes the first 29 AAs at the *N*‐terminus, which are cleaved upon entry to the mitochondrial matrix, reducing the size of CypD from 22 to 19 kDa [176, 177].

**Figure 9:**
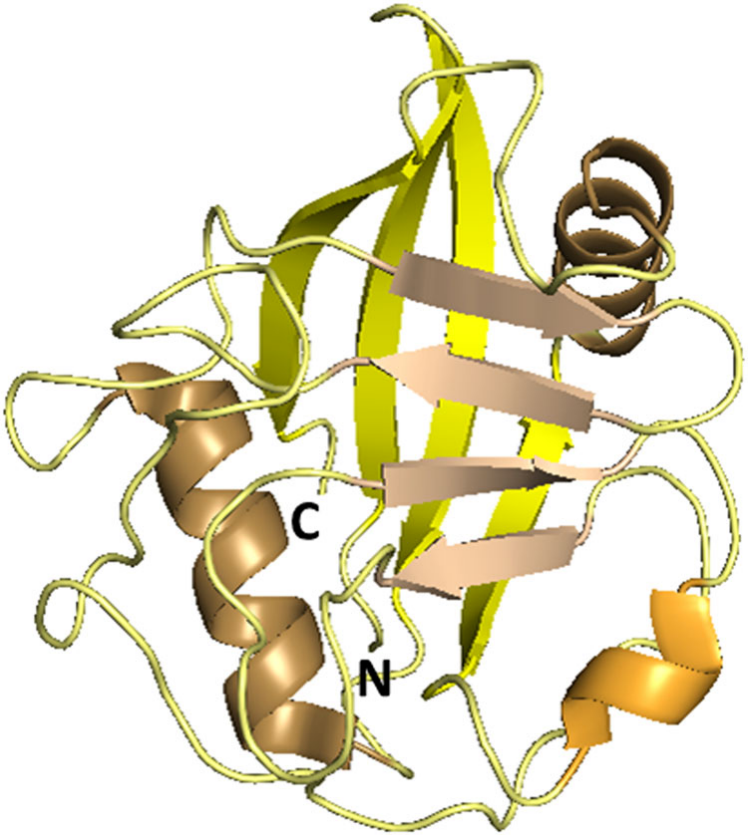
Structure of cyclophilin‐like domain of mitochondrial CypD without the *N*‐terminal mitochondrial targeting sequence (residues 1–29) and the following 14 residues (PDB ID: 2BIT). The *N*‐ and *C*‐termini are indicated. Ribbon representation created using PyMOL software [19]. [Color figure can be viewed at wileyonlinelibrary.com]

In 2007, Kajitani et al. [178] solved the crystal structure of human CypD in complex with CsA. The structure of CypD consists of typical CLD fold (Figure 9). To achieve inhibitor selectivity, the binding sites of CypD were studied [5, 179, 180, 181]. The distinct sites, namely S2 gatekeepers, S1 pocket, and three o΄clock pocket, will be closely discussed later in Section 3.2.

#### 2.5.2 Function of CypD

CypD plays a role in protein folding and maturation, signal transduction, and also acts as a chaperone. The most studied function of CypD is the regulation of the opening of the mitochondrial permeability transition pore (mPTP). Generally, the inner mitochondrial membrane (IMM) is highly impermeable, although specific substrates can penetrate through transporters present in IMM [173, 182, 183]. Cellular stress, damage, or mitochondrial Ca^2+^ overload cause opening of nonspecific pore in the IMM, called mPTP, which enables passage of solutes up to size of 1.5 kDa. The opening of mPTP leads to the loss of IMM potential, uncoupling of oxidative phosphorylation, mitochondrial swelling, rupture of outer mitochondrial membrane and release of apoptogenic proteins from the mitochondrial intermembrane space. These events play an important role in autophagy, apoptosis and necrotic cell death [176, 184]. Thus, CypD is considered a promising drug target in conditions, where excessive apoptotic or necrotic cell death occurs [119, 179, 183, 185, 186, 187, 188, 189, 190, 191, 192, 193].

#### 2.5.3 CypD as a Drug Target

##### 2.5.3.1 IRI

The relationship between mitochondria and cardiac IRI has been extensively studied for decades. There are many detailed reviews on this topic [194, 195, 196]. Reperfusion of ischemic tissues after infarction induces oxidative damage, inflammation, and enlargement of the infarct area. ROS, Ca^2+^ overload, and rapid pH correction induce the opening of mPTP within the first few minutes after reperfusion [197]. CypD, as a key regulator of mPTP, presents a potential target for cardioprotection [196]. The previous studies showed that deletion of CypD decreased infarct size after cardiac IRI in mice [198, 199]. It is important to mention that IRI occurs also in other tissues than heart, such as brain, liver, and kidney.

##### 2.5.3.2 Neurodegeneration

*AD*: Mitochondrial dysfunction is one of the pathophysiological events connected to AD and is considered as a potential target for therapeutic intervention [200]. Restoring mitochondrial function is likely to slow the progression of the disease, in contrast to the currently available therapies that provide mostly palliative treatment. Extensive mitochondrial damage in AD has previously been linked to Aβ toxicity [201, 202, 203, 204, 205]. Despite the uncertain role of Aβ in mitochondria, CypD deficiency caused protection against Aβ‐mediated mitochondrial damage and also improved cognitive functions [189, 205, 206]. CypD inhibition, to suppress the opening of mPTP and protect mitochondria, thus seems promising therapeutic strategy.

*PD*: As mitochondria are especially important for neuronal function, their dysfunction has been associated with neurodegenerative diseases including PD. Besides genetic mutations resulting in mitochondrial dysfunction linked to PD, increased levels of CypD and subsequent opening of the mPTP have been also associated with the disease. The deletion of CypD in PD mouse models showed major effects, such as later disease onset and extended survival [207, 208].

*ALS*: The precise molecular mechanisms behind the pathogenesis of ALS are still poorly understood, which results in no effective therapy for ALS. However, potential therapeutic targets were identified, including mPTP [209, 210]. The increased levels of Ca^2+^ in motor neurons and excitotoxicity link mitochondrial dysfunction and oxidative stress to ALS [211]. It was proposed that elevated Ca^2+^ concentrations are the consequence of activation of plasma membrane glutamate receptors during tetanic stimulation [212]. Excessive stimulation of glutamate receptors ends up with Ca^2+^ overload followed by mPTP opening [213]. Thus, mPTP inhibition via targeting CypD presents a potential therapeutic strategy.

##### 2.5.3.3 Acute Pancreatitis (AP)

AP is a common gastrointestinal disorder caused primarily by gallstones or excessive alcohol intake. Despite the improvement in understanding the key mechanisms that play a role in the development of the disease, there is still no specific drug therapy for AP. In particular, mitochondrial dysfunction is a phenomenon that occurs in the early stages of AP [214]. Different research groups showed that bile acids, ethanol, and fatty acids open the mPTP channel via CypD activation in acinar cells, resulting in mitochondrial depolarization, decreased ATP synthesis, and cell necrosis [214, 215, 216, 217]. Toth et al. [217] studied the non‐immunosuppressive CsA analogue NIM‐811 and provided findings that NIM‐811 is highly effective in pancreatitis models and has no side effects.

##### 2.5.3.4 Nonalcoholic Fatty Liver Disease (NAFLD)

NAFLD is the most common chronic liver disease that starts with hepatic steatosis and can progress to nonalcoholic steatohepatitis (NASH) and further to fibrosis, cirrhosis, and liver cancer. The multifactorial mechanism of NAFLD includes fat accumulation [218], triglyceride de novo lipogenesis [218, 219], and mitochondrial dysfunction represented by impaired hepatic lipid homeostasis and decreased energy transducing capacity [220, 221]. Mitochondrial stress induced by CypD was found to trigger hepatic triglyceride accumulation by excessive mPTP opening and CypD was indicated as a target for NAFLD therapy [222]. Additionally, increased levels of serum CypD were found in diabetic patients with NAFLD and CypD was also suggested as a biomarker for NAFLD diagnosis [223]. A recent study showed that the knock‐down of CypD in the liver can slow down the progression of NASH by suppressing steatosis and inflammatory symptoms, while no significant improvement in liver fibrosis was observed [224].

### 2.6 Cyclophilin E (CypE)

CypE was firstly mentioned in 1996 by Mi et al. [225] when they referred to it as hCyp33, a new nuclear cyclophilin isolated from human T cells. The CypE can be found in both nuclear matrix and nuclear membrane fractions in T cells [226].

#### 2.6.1 Structure of CypE

CypE belongs to the group of multidomain cyclophilins and it contains 301 AA residues. Besides CLD at the *C*‐terminus of the protein, CypE also has an RNA‐binding domain (also known as RNA recognition motif (RRM) or ribonucleoprotein (RNP)) at the *N*‐terminus. In 1996, Mi et al. [225] published the amino acid sequence of CypE derived from the cDNA sequence. The amino acid sequence of CypE can be divided into three main parts. First 84 AA residues of RRM domain, continued by 78 AA residues of linker and 139 AA residues of CLD. The crystal structure of CLD in CypE was firstly resolved as part of the structural genomics initiative, the Structural Genomics Consortium (SGC) [227], and later, in 2010, it was also described by Davis et al. [5] The crystal structure of RRM domain was firstly resolved as part of the RIKEN initiative [228] and later published by three separate groups [7, 229, 230, 231].

**Figure 10:**
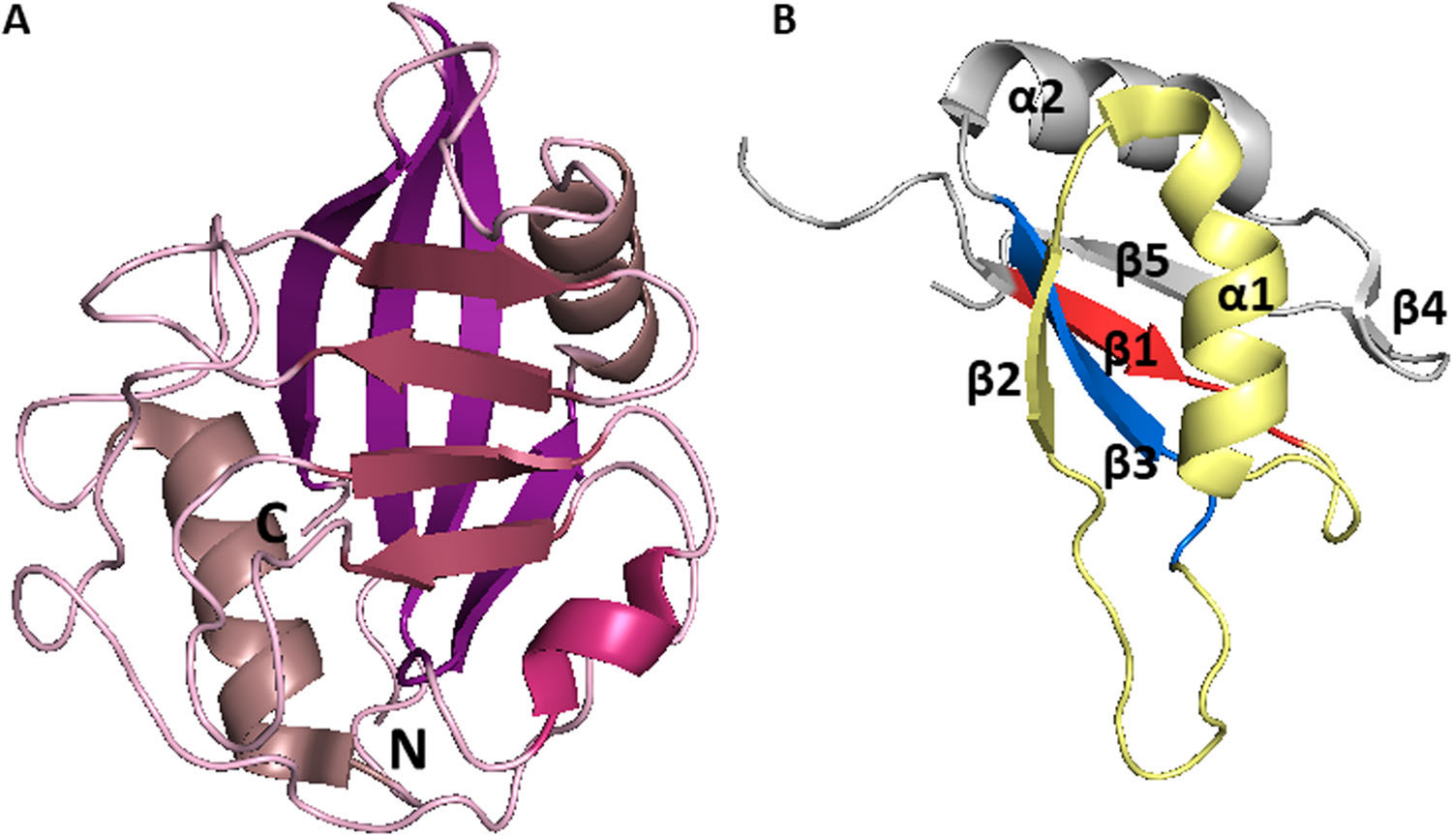
Structure of CypE domains. (A) Structure of CLD of CypE (PDB ID: 2R99) with marked *C‐* and *N‐*termini. (B) Structure of RRM domain of CypE. Submotif RNP‐1 is highlighted in blue, submotif RNP‐2 is highlighted in red and the linker is highlighted in yellow (PDB ID: 3MDF). Ribbon representation created using PyMOL software [19]. [Color figure can be viewed at wileyonlinelibrary.com]

Cyclophilin‐like domain of CypE consists of 139 AAs, is highly conserved, and shows 70% similarity to CypA [232]. Secondary structure features typical cyclophilin‐like fold (Figure 10A). The RRM of CypE (84 residues) consists of two submotifs, RNP‐1 and RNP‐2 connected with a linker of 33 AA residues. The submotif RNP‐1 consists of eight AAs and second submotif, RNP‐2, consists of six AAs [225]. In 2010, Hom et al. [230] determined the secondary structure of RRM domain by X‐ray crystallography. They found that it consists of a five‐stranded antiparallel β‐sheet and two α‐helices (Figure 10B). Helix α1 is placed between β1 and β2 sheets, while α2‐helix is occupying space between β3 and β4 sheets.

#### 2.6.2 Function of CypE

The RRM domain is a typical domain for proteins that play a role in the regulation of RNA processing or translation [225]. CypE was found to be the component of spliceosomes [233, 234].

CypE has been extensively studied for its involvement of in the pathophysiology of leukemia. The *mixed lineage leukemia (MLL)* gene is responsible for chromosomal rearrangements resulting in a development of a different types of acute leukemias (e.g., de novo acute leukemia, therapy‐induced acute myeloid leukemia, and infant leukemia) [231]. It was found that CypE interacts with the third plant homeodomain finger (PHD3) of MLL. The RRM domain interacts directly with the PHD3, whereas CLD interacts with the proline in the MLL protein [7, 229, 230, 231]. This interaction mediates the transition between activation and repression of MLL target genes. CypE reduces the expression levels of MLL target genes by negative regulation of transcriptional activity of MLL [230].

CypE was found to have protective function in association with influenza A virus. Viral ribonucleoprotein complexes (vRNPs) are important components responsible for the replication and transcription of influenza A virus. The virion nucleoprotein (NP) creates a scaffold to hold the vRNPs and has multiple functions in the life cycle of the virus [235, 236, 237]. CypE interacts with the NP of the influenza A virus and thus negatively regulates its replication and transcription.

### 2.7 Cyclophilin G (CypG)

CypG is a multidomain nuclear protein. Throughout the literature, several aliases can be found, for example, Cyp88, SRCyp, or Clk‐associating RS‐cyclophilin (CARS‐Cyp). The gene of CypG (*ppig* gene) is expressed in a variety of tissues and cell types. Two clones of CypG were isolated from mouse T cell cDNA, and then human CypG was firstly isolated from a human thymus cDNA [238, 239].

#### 2.7.1 Structure of CypG

CypG is composed of 754 AAs with a molecular mass of 88 kDa, where 177 AAs build the CLD at the *N*‐terminus [240]. CLD is followed by two nucleolar phosphoproteins of 140 kDa‐related (Nopp140) domains, and a large RS domain at the *C*‐terminus. This part of CypG is highly charged [239], more than 70% of residues from position 180 to 754 are Glu, Asp, Lys, Arg, Ser, or Thr (positively and negatively charged residues alternated regularly) [241]. CypG shares 70% similarity to other RS domain‐containing cyclophilin, CypNK.

**Figure 11:**
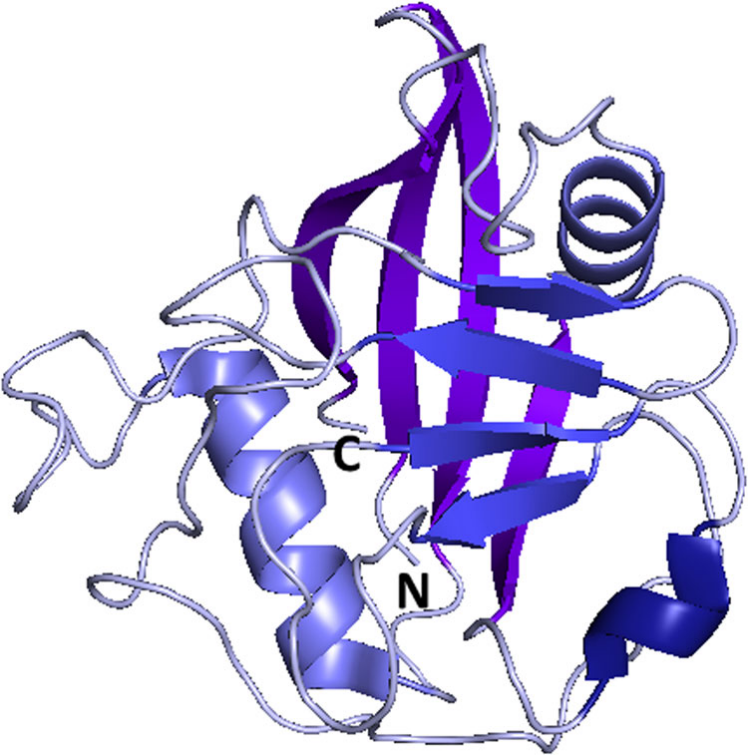
The structure of cyclophilin‐like domain of CypG (PDB ID: 2GW2) with marked *C‐* and *N‐*termini. Ribbon representation created using PyMOL software [19]. [Color figure can be viewed at wileyonlinelibrary.com]

The crystal structure of cyclophilin‐like domain of CypG (Figure 11) was published in 2009 by Stegmann et al. [240] and later also determined by Davis et al. [5] To date, no model for the entire CypG structure has been determined.

#### 2.7.2 Function of CypG

CypG is a nuclear protein associating with the spliceosome. Based on the fact that CypG interacts with Clk (CDC28/cdc2‐like kinase) and possesses the RS domain, it was suggested that CypG plays a role in pre‐mRNA splicing [239, 241]. CypG was found in the spliceosomal C complex and the interaction of its CLD with SNW1 (at α2‐β8 loop), CWC15 (at β3‐β4 loop), and PRPF8 (at 3_10_ helix) was modeled [7, 242, 243, 244]. Nestel et al. [239] suggested the mechanism of CypG function. They proposed that the RS domain or the Nopp140 domain of CypG binds to the SR protein in the cytoplasm and then the formed complex is transported to the nucleus, where it is targeted to speckles. CypG PPIase activity in the speckles helps with the refolding and assembly of splicing factors.

In 2017, Szlavicz et al. [245] described the involvement of CypG in the development of psoriasis. Proliferating keratinocytes produce EDA+ fibronectin, a splice variant of fibronectin overexpressed in psoriasis [246, 247]. CypG was found involved in the fibronectin mRNA maturation processes.

### 2.8 Cyclophilin H (CypH)

CypH was first identified in 1998 by Teigelkamp et al. [248] It is a single‐domain cyclophilin, also known under aliases USA‐Cyp, SnuCyp‐20 or U4/U6‐20K. CypH is located mainly in the nucleus, where it is a part of the spliceosomal [U4/U6·U5] tri‐snRNP (small nuclear ribonucleoprotein particle) complex [249].

#### 2.8.1 Structure of CypH

**Figure 12:**
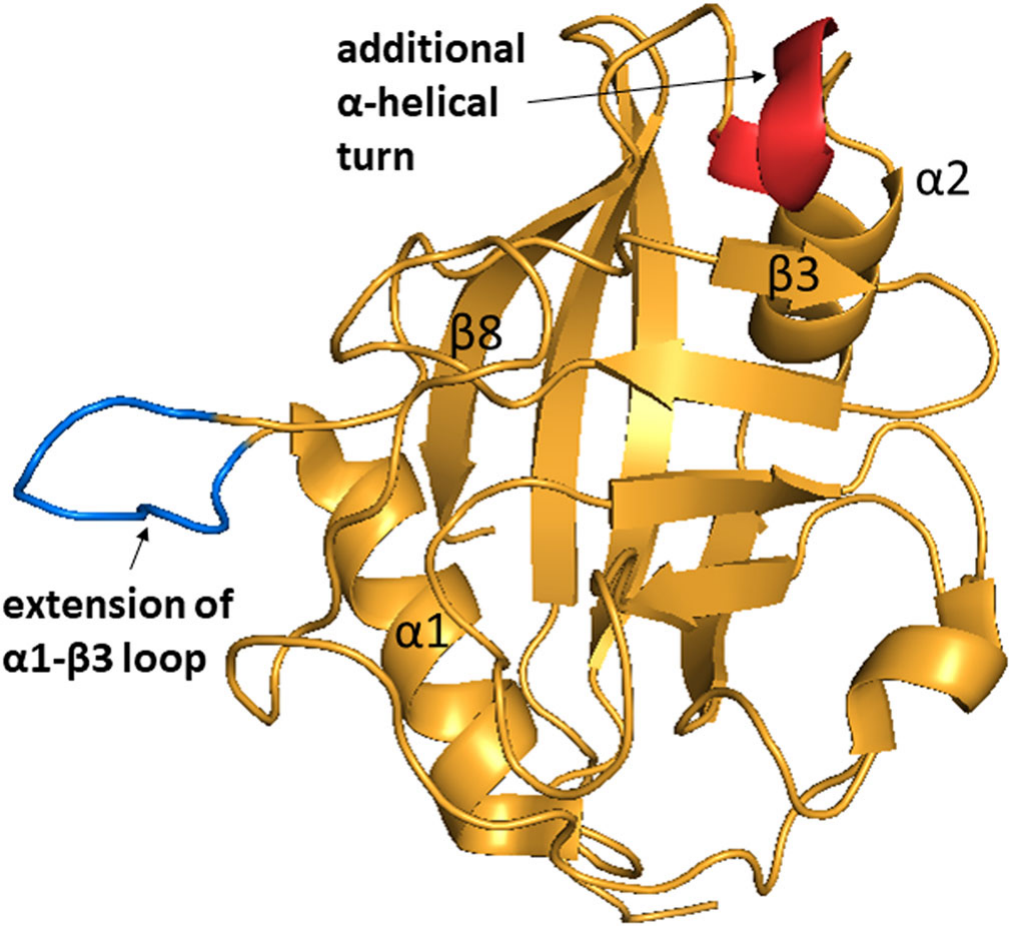
Structure of CypH (PDB ID: 1QOI). Additional short α‐helical turn within in α2‐β8 loop is highlighted in red and the 5 AAs extension of α1‐β3 loop is highlighted in blue. Ribbon representation created using PyMOL software [19]. [Color figure can be viewed at wileyonlinelibrary.com]

The difference between the primary structure of CypH and CypA is in the insertion of additional 13 AAs in CypH [250]. The crystal structure of this 177 AAs long cyclophilin with molecular weight 20 kDa was solved in 2000 by Reidt et al. [250] CypH features the typical structure of CLD. The main difference from is the presence of two short α‐helical turns instead of just one. The second, distinct one, can be found between α2 helix and β8 strand (Figure 12). Another unique structural element is formed by insertion of five AAs in the region of α1‐β3 loop (Figure 12). This region acts as a binding site for protein‐protein interactions.

#### 2.8.2 Function of CypH

It was suggested that the primary role of CypH is in splicing [251]. CypH is a part of spliceosomes, where it interacts with the splicing factor protein PRPF4. Potential interactions with three other near spliceosomal proteins have not been studied yet, but this could provide a better understanding of the role of CypH in splicing regulation [7]. CypH function within the spliceosome was found to be independent of its PPIase activity [251, 252, 253]. Recently, CypH was identified as a prognosis‐related protein in stomach adenocarcinoma [254] and as a susceptibility gene for COVID‐19 in patients with lung adenocarcinoma [255].

### 2.9 Cyclophilin J (CypJ)

CypJ is a single‐domain cyclophilin, also called peptidyl‐prolyl isomerase‐like isoform 3 (PPIL3). It was firstly isolated and characterized by Zhou et al. [256] during the large‐scale sequencing of the human fetal brain cDNA library. CypJ is encoded by *ppil3b* gene. Two different splicing variants can be found in nucleus and cytoplasm [256].

#### 2.9.1 Structure of CypJ

*Ppil3b* gene encodes a 161 AA protein which shares 50% sequence similarity with CypA. An important difference in the AA sequence is the replacement of Trp121 residue in CypA by His110 in CypJ [256]. However, this change does not affect its isomerase activity nor CsA binding.

**Figure 13:**
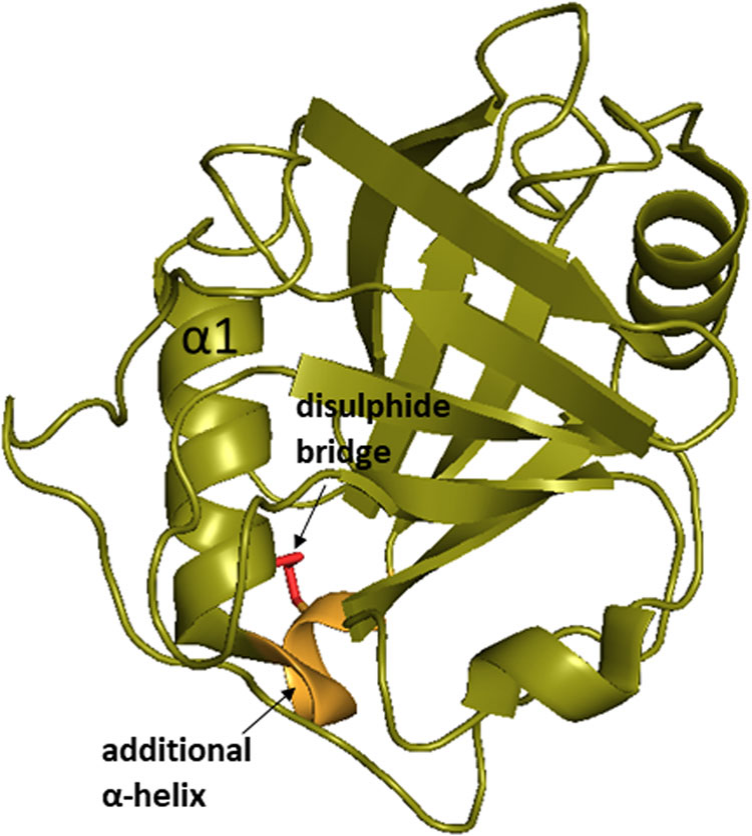
Crystal structure of CypJ (PDB ID: 1XYH). Additional short α‐helix formed by residues Phe17–Thr21, which is followed by standard α1 helix, is highlighted in orange. The unique disulphide bridge between Cys18 and Cys25 is highlighted in red. Ribbon representation created using PyMOL software [19]. [Color figure can be viewed at wileyonlinelibrary.com]

The secondary structure of CypJ was determined by Huang et al. [257] in 2004 and it is similar to other single‐domain Cyps with the typical CLD (Figure 13). Additionally, CypJ possesses a short α‐helix (Figure 13) formed by residues Phe17–Thr21 that is followed by standard α1 helix. A similar short α‐helix can only be seen in the CypA structure. This additional helix also contains a unique disulphide bridge between Cys18 and Cys25 residues, which are not conserved in other Cyps (Figure 13).

#### 2.9.2 Function of CypJ

CypJ belongs to the group of spliceophilins (together with CypE, CypG, CypH, PPIL1, PPIL2, PPWD1, and SDCCAG10) and can be found in spliceosomal B_act_ and C complexes [242].

#### 2.9.3 CypJ as a Drug Target

##### 2.9.3.1 Cancer

Upregulation of CypJ expression was found to increase the cell proliferation and was associated with the multiple malignancies such as liver [258] and stomach cancer [259]. PPIase activity of CypJ was found crucial in regulation of cell cycle through the upregulation of cyclin D1. CypJ promotes cell cycle transition from G1 to S phase resulting in facilitation of tumor growth [258]. In addition, CypJ was found to bind apoptin in cytoplasm of tumor cells, thus preventing apoptin transfer into nucleus and consequent apoptosis of tumor cells [260]. Thus, inhibition of CypJ presents a potential treatment strategy.

### 2.10 Cyclophilin Natural Killer (CypNK)

CypNK is a cytosolic multidomain cyclophilin encoded by the *NK‐TR (natural killer‐tumor recognition)* gene. CypNK, also called Cyp165 or NK‐TR protein, was firstly isolated in 1993 by Anderson et al. [261] from human and mouse cDNA libraries. It was found on the surface of NK cells and is expressed at low levels only in NK cells, making it distinct from other cyclophilins, that are more ubiquitously expressed [261, 262].

#### 2.10.1 Structure of CypNK

The human CypNK consists of 1403 AAs with the molecular weight of 150 kDa. It contains CLD and additional Nopp140 and RS domains. The first 58 AAs of *N*‐terminus form a hydrophobic segment, which is considered to be attached within the cell membrane. This hydrophobic *N*‐terminal part is followed by cyclophilin‐like domain of 192 AA residues, which shares 70% sequence similarity with CypA. The rest of the protein consists of hydrophilic residues, especially serine (more than 20%) and other charged residues (37%, mostly positively charged) [261]. This *C*‐terminal hydrophilic region of the protein contains three Nopp140 repeats and three arginine/serine‐rich (RS) domains [263]. CypNK also shares a 39% homology with other cyclophilin containing the RS domain, CypG.

**Figure 14:**
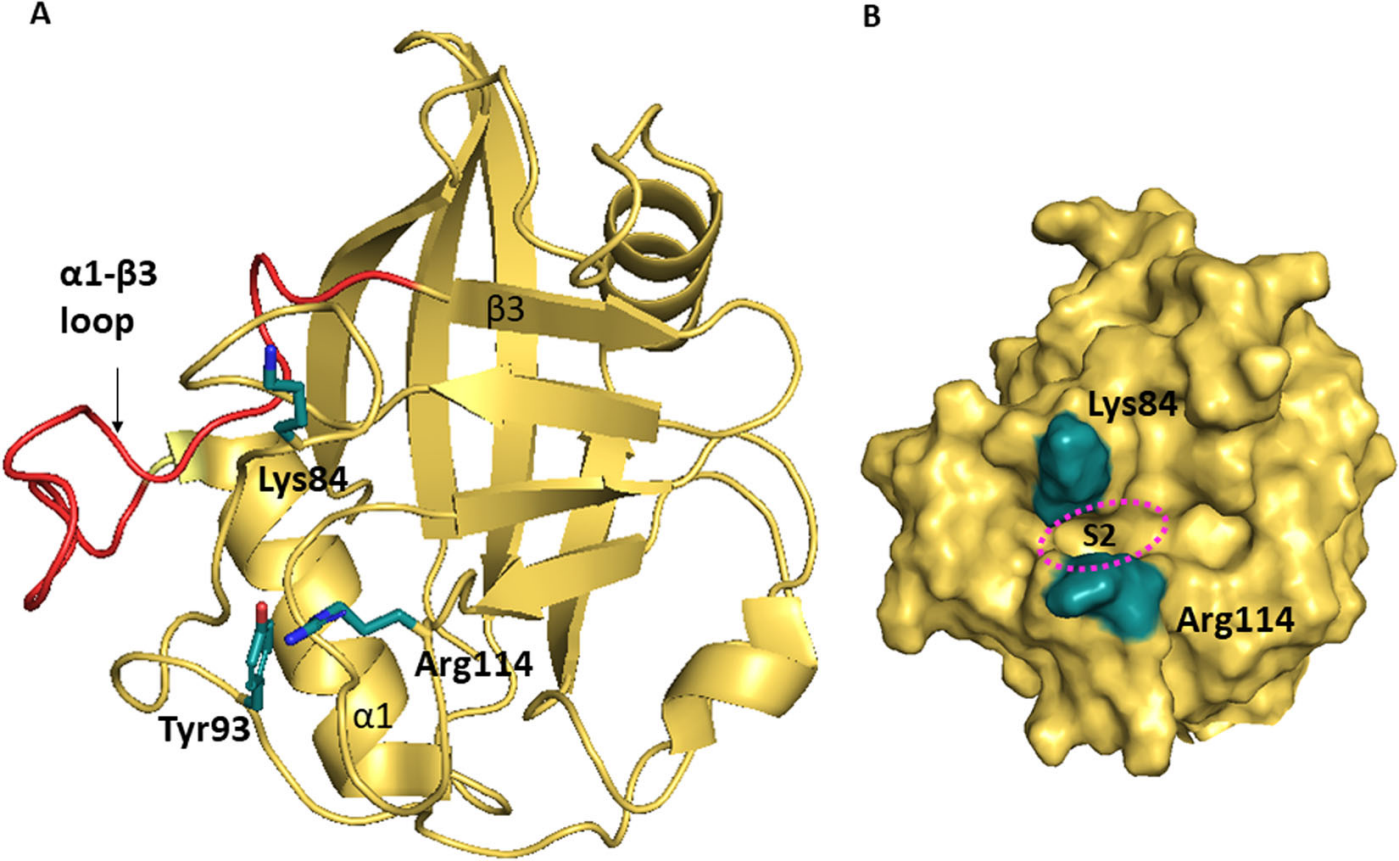
Structure of cyclophilin‐like domain of CypNK (PDB ID: 2HE9). (A) Gatekeeper residues Lys84, Tyr93, and Arg114 with bulky sidechains (in comparison to CypA) restricting access to the S2 pocket are highlighted in green. Elongated α1‐β3 loop is highlighted in red. (B) Surface representation of the narrow gap between gatekeepers Lys84 and Arg114. Ribbon and surface representation created using PyMOL software [19]. [Color figure can be viewed at wileyonlinelibrary.com]

The secondary structure of cyclophilin‐like domain of CypNK was determined in 2010 by Davis et al. [5] It features typical CLD fold similar to other cyclophilins (Figure 14). CypNK differs from CypA in composition of gatekeeper residues, which have bulky sidechains, and thus occlude access to the S2 pocket (Lys84, Tyr93, Arg114; Figure 14A). This extraordinary narrow gap between Lys84 and Arg114 restricts the set of residues that could bind to S2 pocket (Figure 14B). Another difference is in α1‐β3 loop that is longer compared to the loop found in CypA (Figure 14A).

#### 2.10.2 Function of CypNK

CLD of CypNK possesses common protein folding and chaperone activity. The Nopp140 domain acts as a chaperone and imports CypNK into the nucleus, while the CypNK RS domain should participate in the RNA splicing [263, 264, 265]. CypNK plays a role in recognition and lysis of tumor cells by NK cells [266]. Its CLD is thought to be involved in tumor recognition [262]. However, the exact role of CypNK in recognition of tumor cells is still not understood [267].

CypNK was identified as a biomarker of colorectal cancer (CRC) liver metastasis. The expression of the *NK‐TR* gene was lower in CRC with liver metastasis. CypNK was also identified as a negative regulator of progression and metastasis of CRC [268].

### 2.11 Peptidyl Prolyl Isomerase‐Like Isoform 1 (PPIL1)

PPIL1 is a single‐domain cyclophilin located primarily in the nucleus. PPIL1 was firstly isolated from human fetal brain in 1996 by Ozaki et al. [269] It was referred as a novel protein homologous to cyclophilins and called hCypX. PPIL1 was detected in all tested tissues and it was especially abundant in the heart [269].

#### 2.11.1 Structure of PPIL1

**Figure 15:**
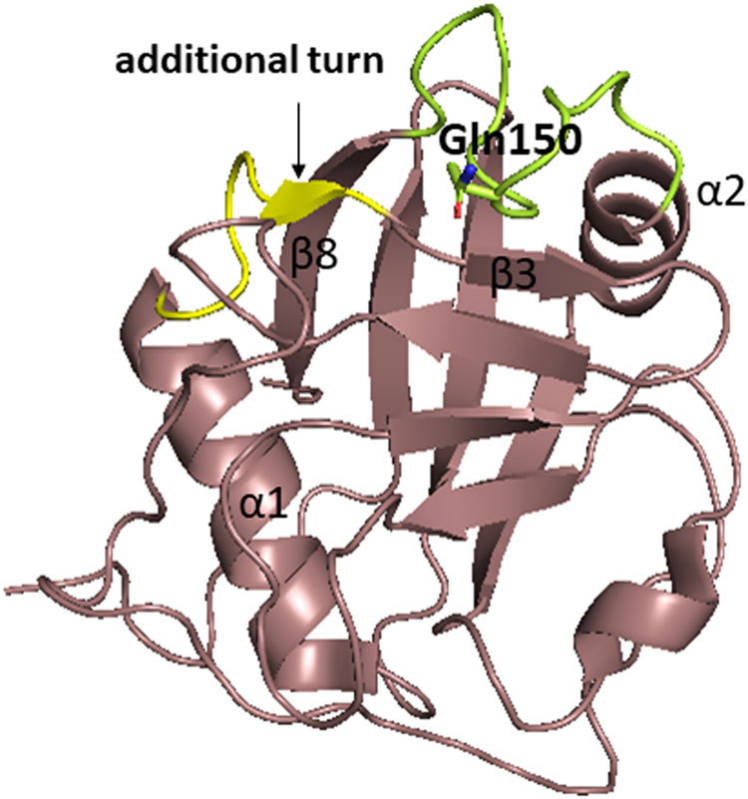
Structure of PPIL1 (PDB ID: 2X7K). The additional turn following α1 helix as a result of the deletion of three AAs (compared to CypA) is highlighted in yellow. α2‐β8 loop with one extra AA (Gln150) which alters its conformation is highlighted in green. Ribbon representation created using PyMOL software [19]. [Color figure can be viewed at wileyonlinelibrary.com]

The primary structure of PPIL1 contains 166 AAs with a molecular weight of 17.9 kDa. The amino acid sequence of PPIL1 shows 42% similarity to CypA [269]. The secondary structure was determined in 2006 by multidimensional heteronuclear NMR spectroscopy [270]. The structure resembles the typical cyclophilin‐like structure (Figure 15). Difference can be found in α1‐β3 loop where 3 AAs are missing, which results in an additional turn following α1 helix. Second difference is insertion of one AA (Gln150) in α2‐β8 loop, which results in altered conformation of the loop compared to CypA [270].

#### 2.11.2 Function of PPIL1

Human PPIL1 is a component of spliceosome. More specifically, it interacts with the SKI‐interacting protein (SKIP) within the 45S and 35S U5 snRNP complexes. The interaction with SKIP probably participates in the activation of the spliceosome [271, 272]. In particular, it was found that the interaction between PPIL1 and SKIP is not inhibited by CsA and does not affect PPIL's enzymatic activity. This indicates that the interaction occurs outside of the enzyme's active site.

#### 2.11.3 PPIL1 as a Drug Target

##### 2.11.3.1 Colorectal Cancer

Obama et al. [273] found that PPIL1 interacts with the cytoplasmic protein stathmin, which controls microtubule dynamics through its phosphorylation. Stathmin promotes microtubule depolymerization by increasing the microtubule catastrophe rate [274]. Thus, overexpression of PPIL1 could regulate microtubule remodeling and it might confer growth‐promoting effect on the cancer cells. Therefore, inhibition of PPIL1 could be a novel therapeutic strategy not only against colon and rectal cancers, but also for other cancer types in which overexpression of PPIL1 has been identified (e.g., cervical, gastric, pancreatic, and chronic myeloid leukemia) [273].

### 2.12 Peptidyl Prolyl Isomerase‐Like Isoform 2 (PPIL2)

PPIL2 is a nuclear multidomain cyclophilin first identified in 1996 by Wang et al. [275] It was first isolated from a human B lymphocytes and the highest expression was detected in thymus, pancreas and testes [275]. There are a few aliases used for PPIL2 throughout the literature, that is, Cyp60, Cyp58, or CYC4.

#### 2.12.1 Structure of PPIL2

**Figure 16:**
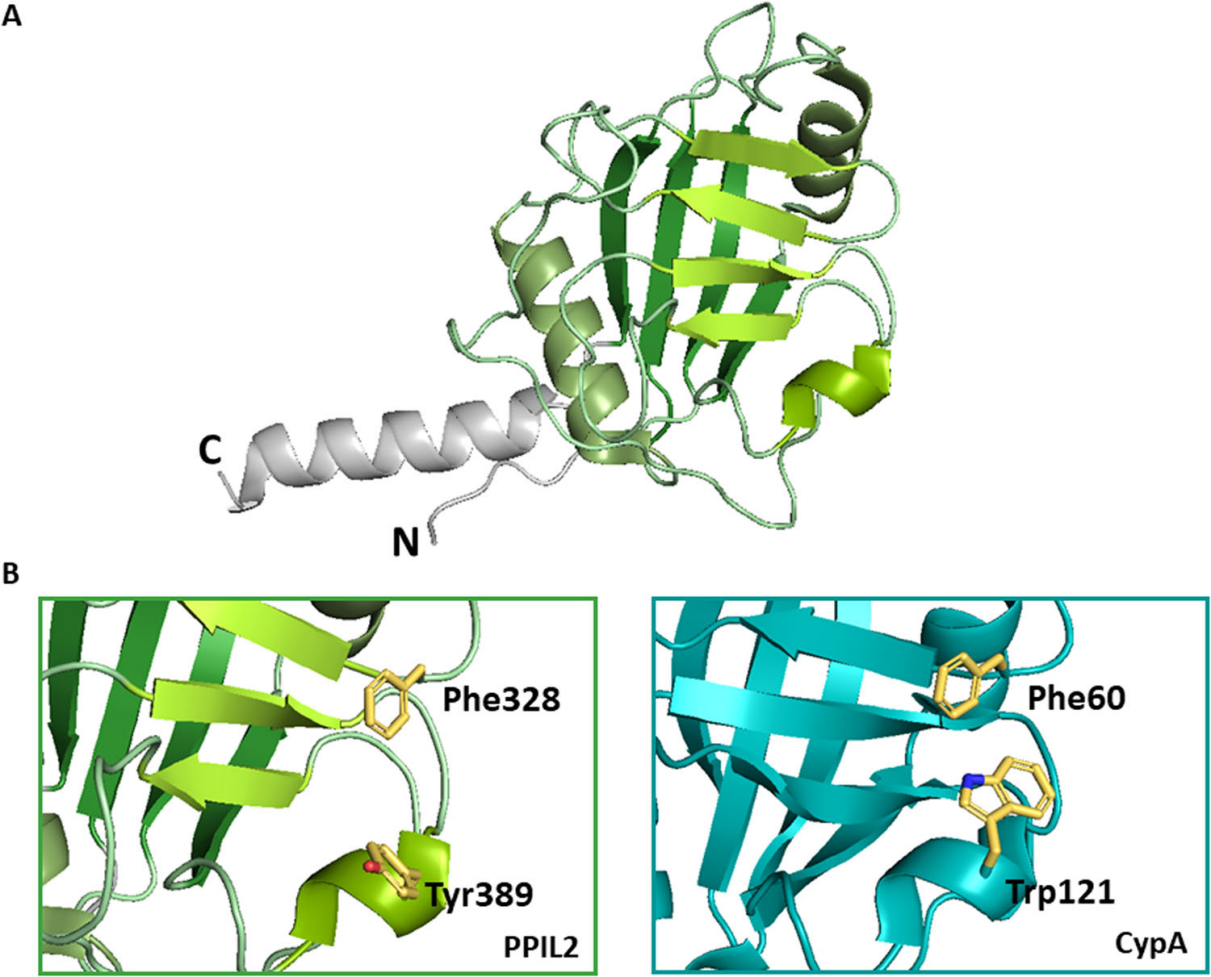
Structure of PPIL2. (A) Structure of CLD of PPIL2 (PDB ID: 1ZKC). (B) Illustration of differences in active site of PPIL2, compared to CypA (PDB ID: 2CPL). Residues Tyr389 of PPIL2 (which corresponds to Trp121 in CypA) and Phe328 (which corresponds to Phe60 in CypA) are highlighted in yellow. Ribbon representation created using PyMOL software [19]. [Color figure can be viewed at wileyonlinelibrary.com]

The structure of PPIL2 consists of 520 AA residues forming the *N*‐terminal U‐box domain and *C*‐terminal CLD with total molecular mass of 60 kDa. The PPIL2 CLD includes 197 AAs and shows roughly 50% similarity to CypA in the region of residues 18–143 of CypA [275]. Crystal structure of CLD was determined and published (Figure 16A) [5]. The substitution of Trp121 in CypA for Tyr389 results in a loss of both isomerase activity and the ability to bind CsA. Residue Tyr389 creates a steric clash with carbonyl group of MLE9 in CsA and also with the residue Phe328 of S1ʹ pocket, which results in turning of the Tyr389 out of active surface (Figure 16B) [5]. U‐box domain belongs to the group of E3 ligase (ubiquitin‐protein ligase) domains [276]. U‐box domain of PPIL2 contains 74 AAs and its structure has not been determined yet [7].

#### 2.12.2 Function of PPIL2

Hatakeyama et al. [277] suggested that the U‐box domain of PPIL2 acts as a functional E3 ligase in the presence of E1 (ubiquitin‐activating enzyme) and E2 (ubiquitin‐conjugating enzyme). They also suggested that U‐box type E3 ligases could be involved in cellular response to stress or to damaged proteins. They could act as quality controls for selection and subsequential polyubiquitination of misfolded proteins for degradation [278]. Later, Davis et al. [5] suggested that non‐active CLD surface could function in spliceosomal complexes or simply bind proline‐containing motifs. PPIL2 was also found to interact with CD147, a signaling receptor for extracellular cyclophilins [122], and thus regulate chemotactic responses in processes like cell‐mediated immunity or inflammation [279, 280].

Recent study suggests that PPIL2 plays a role in DNA repair by involvement in homologous recombination (HR), which is a DNA repair pathway [281]. PPIL2 interacts with proteins related to HR (i.e., ZNF830, CtIP) and is recruited to DNA damage sites. Qiu et al. [281] found that PPIL2 inhibits HR while its downregulation promotes HR. Furthermore, deletion of PPIL2 was found to be associated with congenital heart defects and left ventricular non‐compaction, suggesting the involvement of PPIL2 in cardiac disease [282, 283]. Zhang et al. [284] suggested that decreased expression levels of PPIL2 could identify patients with CAD.

In 2018, Jia and colleagues found PPIL2 to suppress breast cancer invasion and metastasis by altering cell morphology and suppressing epithelial‐mesenchymal transition (EMT) process [285].

#### 2.12.3 PPIL2 as a Drug Target

##### 2.12.3.1 AD

Aβ is a product of β‐amyloid precursor protein (APP) proteolysis by β‐ and γ‐secretases. Espeseth et al. [286] studied the role of ubiquitin ligases in APP processing and identified PPIL2 as a regulator of β‐secretase 1. Knock‐down of PPIL2 decreased β‐secretase 1 levels and overexpression of PPIL2 increased β‐secretase 1 levels. Regulation of β‐secretase 1 levels by PPIL2 was confirmed also in post mortem human brain tissue [287]. These findings indicate that PPIL2 presents a possible target to minimize the production of Aβ [288, 289, 290].

### 2.13 Peptidyl Prolyl Isomerase‐Like Isoform 4

Peptidyl prolyl isomerase‐like isoform 4 (PPIL4), also termed Cyp57, is a multidomain cyclophilin with additional *C*‐terminal RRM domain, similar to CypE. It is located in nucleus and cytoplasm and was first isolated from a human fetal brain [291].

#### 2.13.1 Structure of PPIL4

PPIL4 is a 492 AA long protein with a calculated molecular weight of 57.2 kDa. The structure includes *N*‐terminal cyclophilin‐like domain, RNA recognition motif, a pair of bipartite nuclear targeting sequences, and a lysine rich domain [291]. The CLD consists of 161 AAs with only 36% sequence identity to CypA. Notably, PPIL4 is the only Cyp that has catalytic arginine (Arg55 in CypA) altered (Asn44 in PPIL4), which corresponds with the finding that PPIL4 shows no PPIase activity for standard peptide substrates that are commonly used for assessing PPIase activity [5, 292]. Further, PPIL4 has a Trp121 residue substituted by Tyr118, which is also considered to negatively influence PPIase activity and binding of CsA. RRM domain of PPIL4 contains 79 AA [276].

**Figure 17:**
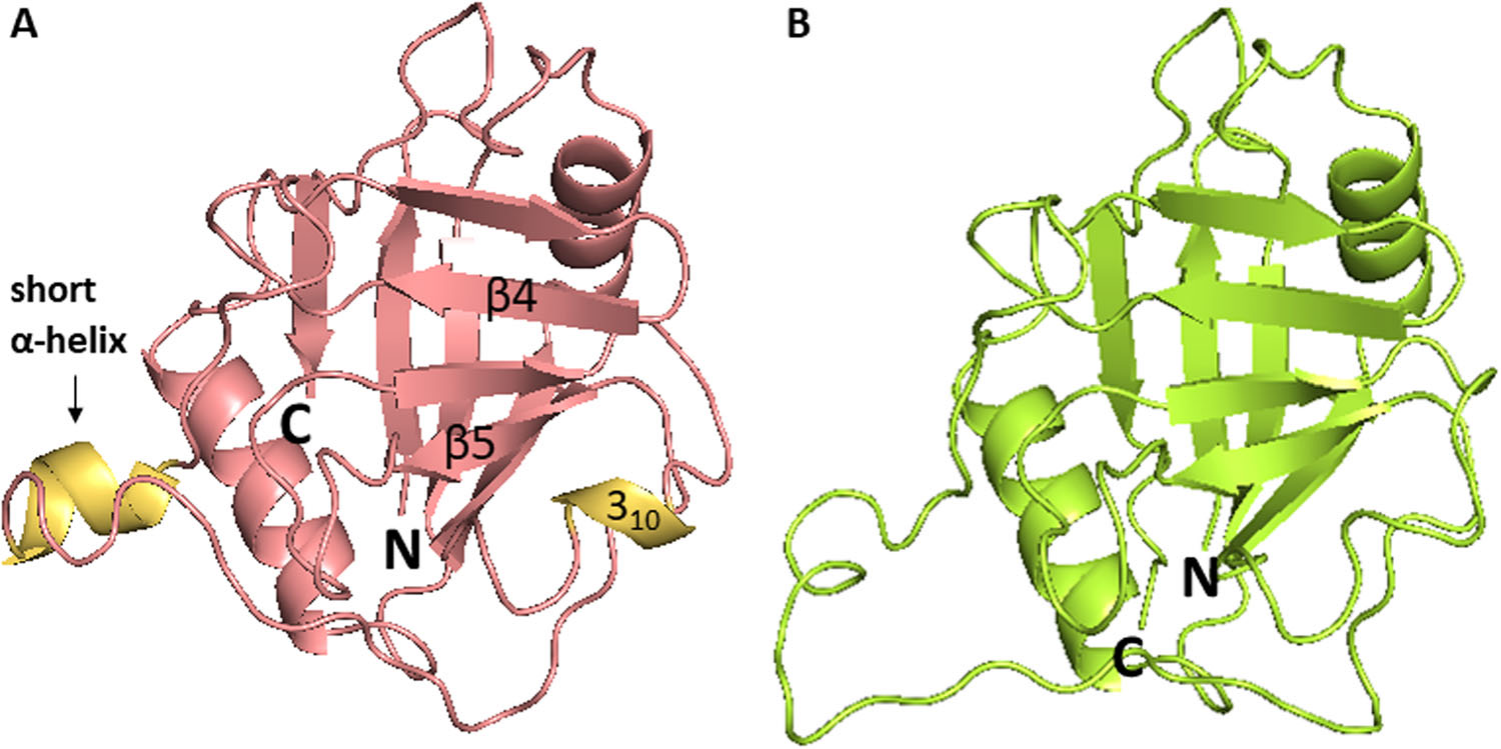
Predicted structure of cyclophilin‐like domain of PPIL4. (A) Structure predicted by the AlphaFold algorithm [293, 294]. Two short helices, that are absent in the second structure, are highlighted in yellow. (B) Structure predicted by Phyre2 algorithm [295] (UniProt accession code: Q8WUA2). Ribbon representation created using PyMOL software [19]. [Color figure can be viewed at wileyonlinelibrary.com]

To date, no crystal structure of PPIL4 was determined, neither for its CLD. Nonetheless, Davis et al. [5] provided the homology model of CLD of PPIL4. Here we provide the predicted structure of CLD of PPIL4 from Alphafold protein structure database [293, 294] (Figure 17A) and a model provided by Phyre2 algorithm [295] (Figure 17B). Both structures feature typical CLD fold. However, the structure predicted by Alphafold shows two short helices (3_10_ helix and additional short α‐helix in β4‐β5 loop) that are absent in the structure by Phyre2.

#### 2.13.2 Function of PPIL4

As previously mentioned, PPIL4 lacks PPIase activity and binding affinity for CsA [5]. Human PPIL4 was identified as a component of the spliceosomal B complex [296] and was associated with rheumatoid arthritis, where it could influence the development of inflammation [297]. Recently, PPIL4 was found to play a role in brain angiogenesis and mutations in its gene were implicated in pathogenesis of intracranial aneurysm, a disorder leading to subarachnoid haemorrhage [298].

### 2.14 Peptidyl Prolyl Isomerase‐Like Isoform 6 (PPIL6)

PPIL6 is a poorly characterized cyclophilin mentioned only in a few publications [5, 7, 276]. According to UniProtKB [299] its subcellular localization is in the cytosol, Golgi, and nucleus.

#### 2.14.1 Structure of PPIL6

**Figure 18:**
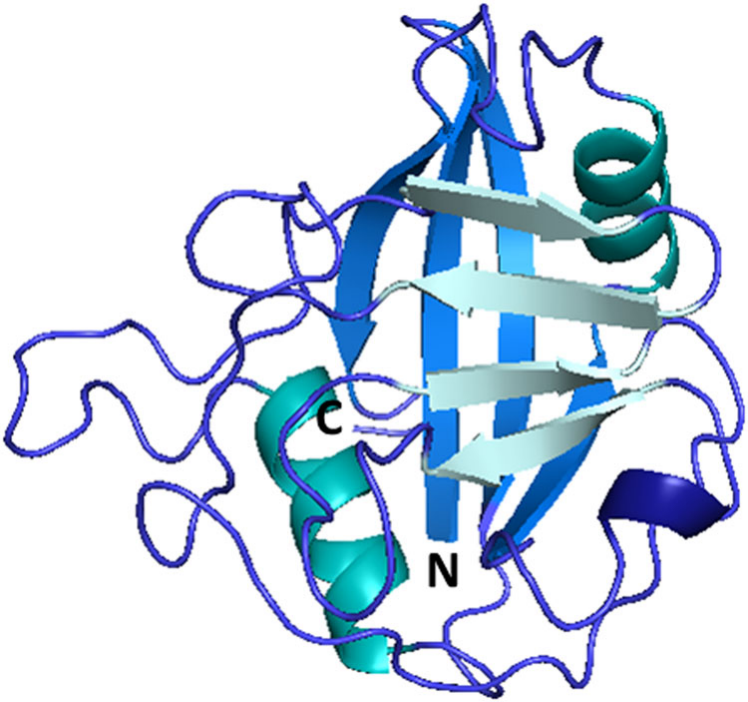
Predicted structure of CLD of PPIL6 by AlphaFold algorithm [293, 294] (UniProt accession code: Q8IXY8). *C‐* and *N‐*termini are marked. Ribbon representation created using PyMOL software [19]. [Color figure can be viewed at wileyonlinelibrary.com]

Human PPIL6 gene was firstly identified by Mammalian gene collection program team in 2002 [300]. PPIL6 consists of 311 AAs of which 164 AAs create CLD of predicted structure depicted in Figure 18 [276]. In 2010, Davis et al. [5] provided the model for PPIL6 CLD by using homology modeling in Phyre algorithm [301]. They also found that PPIL6 does not bind CsA and has no isomerase activity, probably due to structural differences, particularly the substitution of Trp121 in CypA with Tyr266 in PPIL6.

#### 2.14.2 Function of PPIL6

There is no information on the putative physiological functions of PPIL6. Most likely, PPIL6 lacks PPIase catalytic activity and does not bind CsA. In 2015, Schiene‐Fischer [276] included PPIL6 in a multi‐domain cyclophilins group. It was referred as Cyp35 and only mentioned that its isomerase activity has not been shown yet. Later in 2018, Rajiv and Davis [7] briefly mentioned PPIL6 as a nuclear cyclophilin.

### 2.15 Peptidyl‐Prolyl Isomerase Containing WD40 Repeat (PPWD1)

PPWD1 is a multidomain nuclear cyclophilin, also termed Cyp73 or KIAA0073. PPWD1 was first cloned in 1994 [302] and later in 2003 identified as a part of spliceosome C complex [303].

#### 2.15.1 Structure of PPWD1

Human PPWD1 consists of 646 AAs with a molecular weight of 73 kDa. PPWD1 contains the *N*‐terminal WD40 domain and *C*‐terminal CLD. WD40 repeats were firstly described as regions formed by repetitive sequence motifs of WD (Trp‐Asp) dipeptide about 40 AAs long [304]. In PPWD1 can be found four such WD40 repeats [276]. CLD consists of 176 AA residues with 60% similarity to CypA [305].

**Figure 19:**
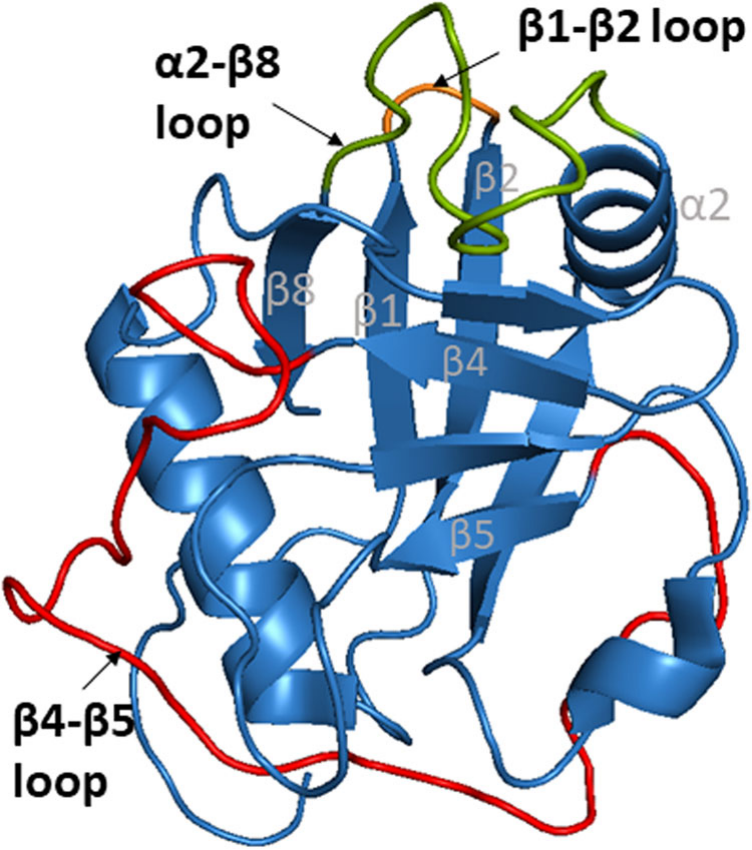
Structure of cyclophilin‐like domain of PPWD1 with diverse α2‐β8, β1‐β2, and β4‐β5 loops highlighted in green, orange, and red, respectively (PDB ID: 2A2N). Ribbon representation created using PyMOL software [19]. [Color figure can be viewed at wileyonlinelibrary.com]

The crystal structure of CLD of PPWD1 was determined in 2008 by Davis et al. [305] It consists of typical CLD fold (Figure 19) similar to the structure CypA. The main differences are in the α2‐β8, β1‐β2, and β4‐β5 loops (Figure 19). In α2‐β8 loop, there is only one AA identical to those in CypA. PPWD1 has 5 AAs deletion in β1‐β2 loop, however, the consequences of the shorter loop are not known. β4‐β5 loop differs in six AA residues in comparison to CypA, which could influence the substrate specificity. The secondary structure of the WD40 domain of PPWD1 has not been determined yet.

#### 2.15.2 Function of PPWD1

PPWD1 acts as a functional isomerase against standard peptide substrates. Interestingly, a peptide that interacts with the PPWD1 was also identified, but it is not a substrate for isomerization. As a component of spliceosome, isomerase domain of PPWD1 probably plays a role in spliceosomal assembly and activity, or serves as a signal transduction factor [305]. WD40 domain of PPWD1 mediates signal transduction, transcriptional regulation and apoptosis [306, 307]. PPWD1 was found to be highly abundant only in spliceosomal C complex [242, 243]. PPWD1 interacts with the other parts of C‐complex, and thus potentially stabilizes the complex and regulates its activity [308].

PPWD1 acts as a marker of gastroenteropancreatic neuroendocrine tumors (GEP‐NETs). It was shown that primary NETs from the pancreas, small intestine and stomach have different expression profiles of three genes, namely *CD302*, *ABHB14B*, and *PPWD1* [309]. The study showed that downregulation of *PPWD1* gene expression indicates the pancreas as the primary tumor tissue [310].

Several studies have found that lncRNAs (long noncoding RNAs) and miRNAs (small noncoding RNAs) together with PPWD1 are involved in tumorigenesis [311, 312, 313, 314, 315]. Han et al. [316] were the first to report the tumor suppressor role of PPWD1 in cervical cancer. The RP11‐284F21.9/miR‐769‐3p/PPWD1 axis regulates proliferation, migration and invasion of cervical cancer cells, and thus represents a promising biomarker and therapeutic target for cervical cancer. In addition, PPWD1 was shown to be involved in a lung cancer. Recent studies showed participation of miRNAs in the tumorigenesis and lung cancer progression [317, 318]. MiR‐629‐5p was found to promote cell migration and invasion in lung adenocarcinoma (LUAD) through inhibition of PPWD1 expression. According to this finding, overexpression of PPWD1 decreased the promoting effect of miR‐629‐5p on cell migration and invasion in LUAD cells [319].

Postmenopausal osteoporosis (PMO) is the most common type of osteoporosis caused by estrogen deficiency, which results in an imbalance in bone formation and resorption [320]. In 2019, Qian et al. [320] studied the genes involved in PMO to identify potential biomarkers. The bioinformatics‐based approach indicated that PPWD1 is a possible candidate. PPWD1 was proposed to affect bone metabolism by regulating fatty acid metabolism, however, this finding has to be further investigated.

### 2.16 Ran‐Binding Protein 2 ANBP2

RANBP2 is a large protein with calculated molecular weight of 358 kDa belonging to the group of multidomain cyclophilins. RANBP2 was firstly mentioned in 1995, by Wu et al. [321] when they obtained its primary structure. RANBP2 was found to be localized at the cytoplasmic fibers of the nuclear pore complex (NPC), and thus was named nucleoporin of 358 kDa (Nup358). Other aliases that can be found in the literature are Cyp358 and RanBP2.

#### 2.16.1 Structure of RANBP2

The primary structure of RANBP2 consists of 3224 AAs, which makes it the largest known cyclophilin. Wu et al. [321] described the structure of RANBP2 that contains *N*‐terminal leucine‐rich domain comprised of three TPRs, four Ran‐binding domains (RBDs), eight zinc finger (ZF) domains and *C*‐terminal CLD. The cyclophilin‐like domain of RANBP2 shows 67% sequence similarity to CypA [322]. Later, in 2002, Pichler et al. [323] showed that RANBP2 also contains E3 SUMO (small ubiquitin‐related modifier) ligase domain. The particular domains are connected with regions of phenylalanine‐glycine (FG) repeats throughout the protein [324].

**Figure 20:**
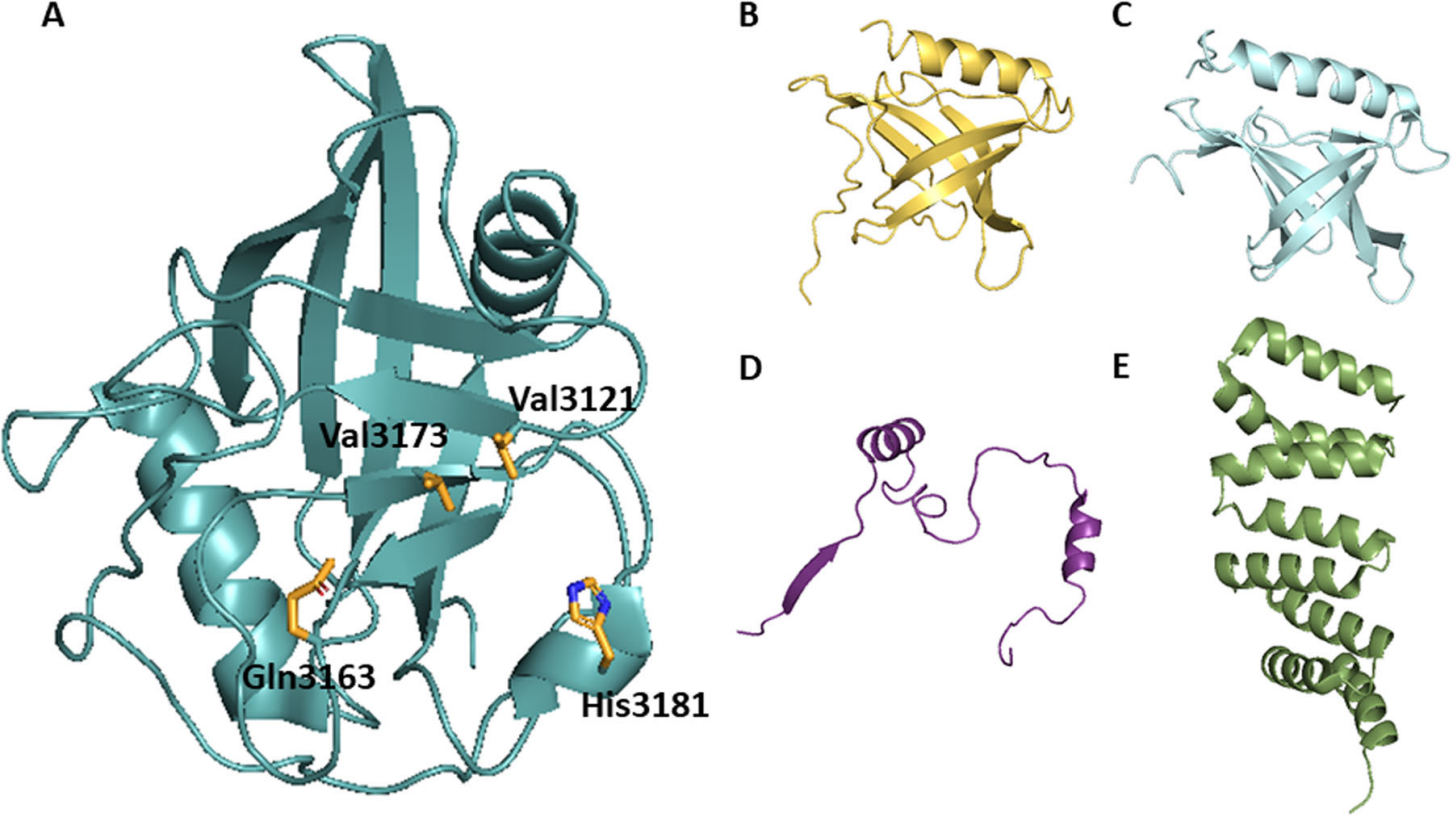
Structures of the characterized domains of RANBP2. (A) Structure of cyclophilin‐like domain with typical CLD fold with active site residues that are diverse from CypA highlighted in orange (PDB ID: 4I9Y). (B) Structure of RBD1 domain, which forms an up‐and‐down β‐barrel with two orthogonal β‐sheets consisting of four and two β‐strands and one α‐helix on the top (PDB ID: 1RRP). (C) Structure of RBD2 domain, which contains two orthogonal antiparallel β‐sheets consisting of three and four β‐strands forming an up‐and‐down β‐barrel and one α‐helix sitting on top (PDB ID: 1XKE). (D) Structure of the IR1‐M part of the E3 SUMO ligase domain, which forms a structure with one β‐strand and two α‐helices (PDB ID: 1Z5S). (E) Structure of *N*‐terminal domain, which forms right‐handed twisted sheet consisting of eight α‐helices stacked in a zig‐zag arrangement (PDB ID: 4DA0). Ribbon representation created using PyMOL software [19]. [Color figure can be viewed at wileyonlinelibrary.com]

So far, structures of *N*‐terminal TPR domain, SUMO E3 ligase domain, two Ran‐binding domains (RBD1, RBD2) and *C*‐terminal CLD of RANBP2 were determined [324, 325, 326, 327, 328]. CLD consists of 162 AAs that form a typical cyclophilin‐like fold, however, it shows differences from active site of CypA. Active site residues Met61, Ala103, Phe113, and Trp121 in CypA are substituted for Val3121, Gln3163, Val3173, and His3181 in RNBP2 (Figure 20A), resulting in different interactions with substrates and inability to bind CsA [328]. RBD1 has the β‐barrel topology of a pleckstrin homology domain fold. It forms an up‐and‐down β‐barrel with two orthogonal β‐sheets consisting of four and two β‐strands and one α‐helix on the top (Figure 20B). The main difference from the fold of the pleckstrin homology domain is that the second β‐sheet consists of two β‐strands instead of three [325]. RBD2 forms a pleckstrin homology fold with two orthogonal antiparallel β‐sheets consisting of three and four β‐strands forming an up‐and‐down β‐barrel and one α‐helix sitting on top (Figure 20C) [324, 327]. E3 SUMO ligase domain consists of two fragments of approximately 50 AAs (IR1 and IR2) separated by the M‐domain of 25 AAs. IR1 and IR2 share 40% identity with each other. IR1‐M domain forms structure with one β‐strand and two α‐helices (Figure 20D) [326]. *N*‐terminal domain (NTD) of 830 AAs forms a slightly curved, right‐handed twisted sheet consisting of eight α‐helices stacked in a zig‐zag arrangement (Figure 20E). NTD is primarily composed of three TPR repeats, each forming a pair of antiparallel α‐helices.

#### 2.16.2 Function of RANBP2

CLD of RANBP2 possesses PPIase activity, however, with a much lower efficiency than CypA [328]. Finding that RANBP2 interacts with proteasome suggests that it could modulate the activity of the ubiquitin‐proteasome system and protein biogenesis via its CLD [329, 330].

PPIase and chaperone activity of CLD of RANBP2 regulates the proteostasis of the signal transducer and activator transcription 3 and 5 (STAT3/STAT5), heterogenous nuclear ribonucleoprotein A2B1 (hnRNPA2B1), and M‐opsin. Regulation of these substrates is very important due to its involvement in diseases like cancer, neurodegeneration, inflammation, multisystem proteinopathies, ALS, cone photoreceptor neuron dystrophy, and color blindness [331, 332, 333, 334, 335, 336].

Ferreira and colleagues [329, 337, 338, 339] studied the function of bovine RANBP2, which is a counterpart of human and murine RANBP2, as a novel retinal cyclophilin in photoreceptor cells. They found that Ran‐binding domain 4 (RBD4) and CLD act together as chaperones for the red/green opsin molecule. CLD enhances and stabilizes the interaction of red/green opsin with RBD4, probably through its isomerization activity [339].

The Ran‐binding domain of RANBP2 interacts with Ran (RAs‐related nuclear protein, GTP‐binding nuclear protein) in a GTP‐dependent manner. Ran‐GTPase is crucial for both nuclear import and export [340]. Specifically, the zinc finger domain was found to be associated with the nuclear export processes through its interaction with the nuclear export factor, exportin‐1 [340]. To facilitate nucleocytoplasmic trafficking, RANBP2 acts as a SUMO E3 ligase and localizes SUMO1‐modified RanGAP1 and Ubc9 (ubiquitin conjugating enzyme 9) at the NPC [341].

Several studies reported mutations in RANBP2 gene to be linked to the acute necrotizing encephalopathy (ANE), however, its pathogenic mechanism is unclear [342, 343, 344]. Jiang et al. [345] discussed possible roles of RANBP2 in acute necrotizing encephalopathy type 1 (ANE1), where among others they mentioned the theory that mutations in RANBP2 trigger a cytokine storm, which results in elevated levels of pro‐inflammatory cytokines.

RANBP2 was previously connected to Parkinson's disease. The *parkin* gene is a causative agent of early onset familial form of PD. It encodes a ubiquitin‐protein ligase E3 and was identified as a modulator of the RANBP2 enzymatic activity. It was suggested that parkin modulates RANBP2 enzymatic activity via ubiquitination followed by degradation, which may contribute to cell death in PD [346].

#### 2.16.3 RANBP2 as a Drug Target

##### 2.16.3.1 Viral Infections

RANBP2 was reported to be involved in the life cycle of several viruses such as herpes simplex viruses, adenoviruses, vaccinia virus, papillomaviruses, SARS‐Cov‐2, human rhinovirus, HCV, JEV, influenza, and HIV‐1 [347, 348, 349, 350, 351, 352, 353, 354, 355]. It was suggested that RANBP2 interacts either with the viral proteins or antiviral host factors, and thus affects viral infection process [345]. CLD of RANBP2 was found to interact with the CypA‐binding loop of the HIV‐1 capsid (similarly to CypA), and thus facilitates import of HIV‐1 preintegration complex to nucleus and promotes viral infection [345].

##### 2.16.3.2 Cancer

RANBP2 was linked to the chromosomal missagregation during mitosis, which is a common feature of many tumors. It was proposed that depletion of RANBP2 causes mitotic catastrophe resulting in cell death, and therefore RANBP2 may play an important role in mitotic progression and chromosomal segregation [356]. These findings could result in the discovery of the new cancer therapies.

### 2.17 SDCCAG10

Serological defined colon cancer antigen 10 (SDCCAG10) is a nuclear spliceosomal cyclophilin also called CWC27, Cyp54, or NY‐CO‐10. It is encoded by *CWC27* gene and belongs to the group of multi‐domain cyclophilins.

#### 2.17.1 Structure of SDCCAG10

SDCCAG10 consists of *N*‐terminal CLD and an elongated, solvent‐exposed *C*‐terminus. Two isoforms of SDCCAG10 were described with the difference in the length of the *C*‐terminal domain, Q6UX04‐1 (472 AAs, 54 kDa) and Q6UX04‐2 (390 AAs, 44 kDa). The CLDs of both isoforms are identical and include 156 AA residues [357]. The crucial difference from CypA is the substitution of Trp121 (in CypA) with Glu122, which was associated with the loss of PPIase activity and CsA binding affinity. However, it was suggested that SDCCAG10 could bind proline‐containing peptides without catalysis [5].

**Figure 21:**
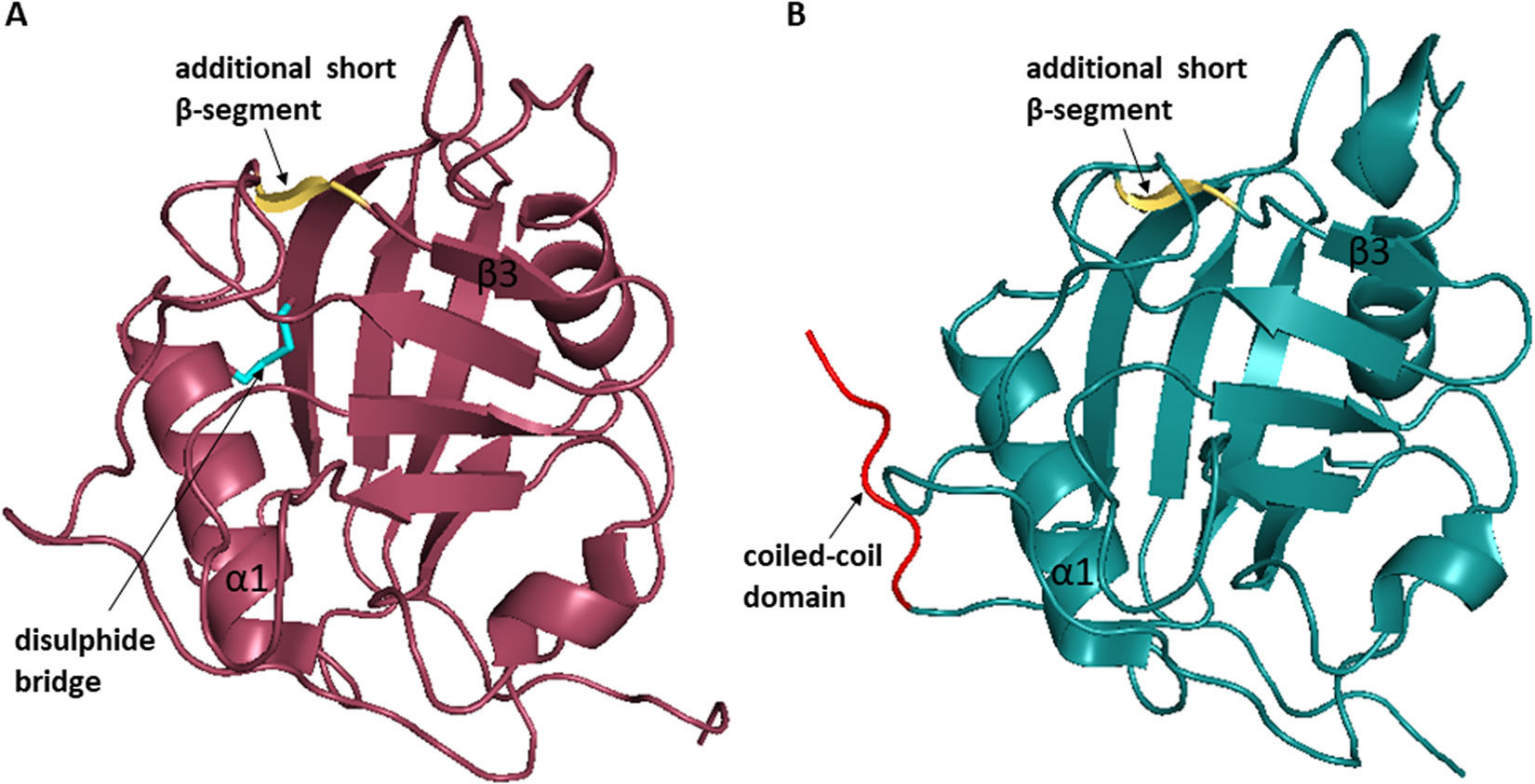
Structures of the cyclophilin‐like domain of SDCCAG10. (A) Structure determined by Davis et al. [5] (PDB ID: 2HQ6). Additional short β‐segment present in the α1‐β3 loop is highlighted in yellow. Disulphide bond between Cys44 and Cys164 is highlighted in light blue. (B) Structure determined by Ulrich et al. [357] (PDB ID: 4R3E). Additional short β‐segment present in the α1‐β3 loop is highlighted in yellow. Part of a *C*‐terminal region called the coiled‐coil domain (residues Asp173–Glu178) is highlighted in red. Ribbon representation created using PyMOL software [19]. [Color figure can be viewed at wileyonlinelibrary.com]

Two crystal structures of CLD of SDCCAG10 were published. First, the structure was determined by Davis et al. [5] (Figure 21A) and later by Ulrich and Walh [357] (Figure 21B). The CLD features the common cyclophilin‐like fold. In addition, SDCCAG10 contains a short β‐segment in the α1‐β3 loop. The CLD is followed by a partially α‐helical elongated *C*‐terminus, also called the coiled‐coil domain, which is likely to be solvent‐exposed [357]. There are subtle differences between the two determined crystal structures. The structure determined by Davis et al. [5] includes residues 3–172, while the structure by Ulrich and Wahl [357] includes residues 6–178. Notably, the latter mentioned includes the residues Asp173–Glu178, which are a part of the coiled‐coil domain (Figure 21B). Additionally, this structure does not contain disulphide bond between Cys44 and Cys164 present in the structure by Davis et al. [5] (Figure 21).

#### 2.17.2 Function of SDCCAG10

SDCCAG10 was found to be associated with spliceosomal complexes and its CLD was modeled into three of them, namely the mature B_act_ complex, the late B_act_ complex and the C complex [7, 242, 358, 359]. The function of SDCCAG10 in splicing is unclear; however, it was suggested that its ability to bind proline‐containing sequences may play a role [357]. In addition, mutations in *CWC27* gene, which encodes SDCCAG10 protein, have been associated with the spectrum of spliceosomopathies including retinal degeneration, brachydactyly, craniofacial defects, short stature, and neurological defects [360].

#### 2.17.3 SDCCAG10 as a Drug Target

##### 2.17.3.1 Bladder Cancer

Bladder cancer is a common type of malignancy with aetiology including both genetic and environmental factors [361, 362]. The overexpression of SDCCAG‐10 was found in bladder cancer, and it was suggested that it induces the cell proliferation and suppresses apoptosis of bladder cancer cells. However, the exact mechanism remains undefined and further investigation is needed [363].

## 3 Inhibition of Cyclophilins

Inhibition of cyclophilins is considered a potential treatment strategy in a variety of diseases. However, there are no cyclophilin inhibitors in clinical practice to date. The only exception is CsA, which is, however, not used for its ability to inhibit PPIase activity of cyclophilins, but for its immunosuppressive function based on a distinct mechanism of action [364]. A major challenge presents selective inhibition of particular members of the cyclophilin family. Here, we provide an overview of Cyp inhibitors with special focus on development of selective molecules.

### 3.1 Nonselective Cyclophilin Inhibitors

**Figure 22:**
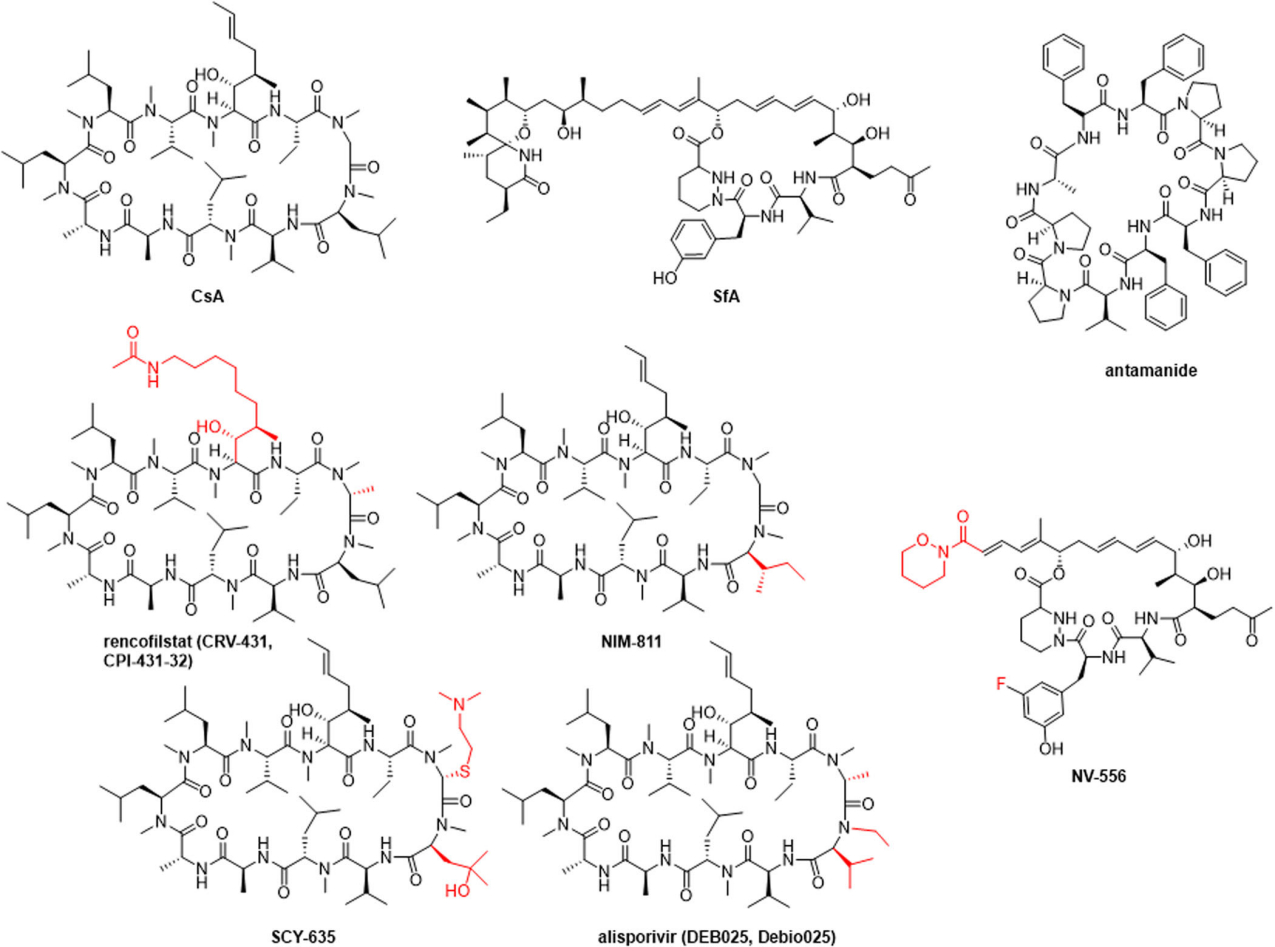
Chemical structure of natural macrocyclic cyclophilin inhibitors CsA, SfA, antamanide, and their analogues. CsA derivatives: NIM‐811, SCY‐635, alisporivir (DEB025, Debio025), and rencofilstat (CRV‐431, CPI‐431‐32); SfA derivative NV‐556. Modifications to parent natural compounds are highlighted in red. [Color figure can be viewed at wileyonlinelibrary.com]

Natural products, such as CsA, sanglifehrin A (SfA), and antamanide [365] (Figure 22) are potent Cyp inhibitors, however, their immunosuppressive effect [366], low selectivity [5], and troublesome drug‐like properties (low oral bioavailability, nephrotoxicity, hepatotoxicity, poor solubility, drug–drug interactions, etc.) [367, 368, 369] limit their applicability. To improve the mentioned problems, non‐immunosuppressive derivatives of CsA and SfA (Figure 22) have been developed. Several nonimmunosuppressive CsA derivatives, such as NIM‐811, SCY‐635, alisporivir (DEB025, Debio025), and rencofilstat (CRV‐431, CPI‐431‐32) (Figure 22) have been subjected to preclinical or clinical trials [367]. SCY‐635 and alisporivir underwent clinical trials for HCV treatment, however they exhibited undesired side‐effects [367, 370, 371]. Recently, clinical study of alisporivir for the treatment of infections due to COVID‐19 was completed. However, its results have not been published yet. Clinical study of rencofilstat for the treatment of NASH and advanced liver fibrosis is currently undergoing [372]. Another noteworthy non‐immunosuppressive CsA derivative was reported by Malesevic et al. [373] that exclusively inhibited extracellular cyclophilins due to its cell‐impermeable character. From SfA derivatives, NV‐556 (Figure 22) presents a promising candidate for preclinical studies [364, 374, 375, 376].

The inherent drawback of above mentioned natural inhibitors (and their analogues) is their poor physical‐chemical properties owing to their macrocyclic (peptidic or in case of SfA peptido‐macrolidic) structure, which hampers their pharmacokinetic profile (low oral bioavailability, low penetration into certain tissues etc [377, 378]). From this point of view, synthetic small‐molecule inhibitors seem to be more promising. Yet, none of these compounds has advanced to clinical trials so far. To summarize knowledge of all small‐molecule cyclophilin inhibitors is beyond the scope of this article and for more information we refer to other review articles, for example, nonimmunosuppressive Cyp inhibitors [364], small‐molecule inhibitors of CypD [193], or CypA inhibitors [58]. In the following text we will focus only on the selective inhibitors targeting particular members of cyclophilin family and the medicinal chemistry aspect of their development.

### 3.2 Differential Sites of Cyclophilins

**Figure 23:**
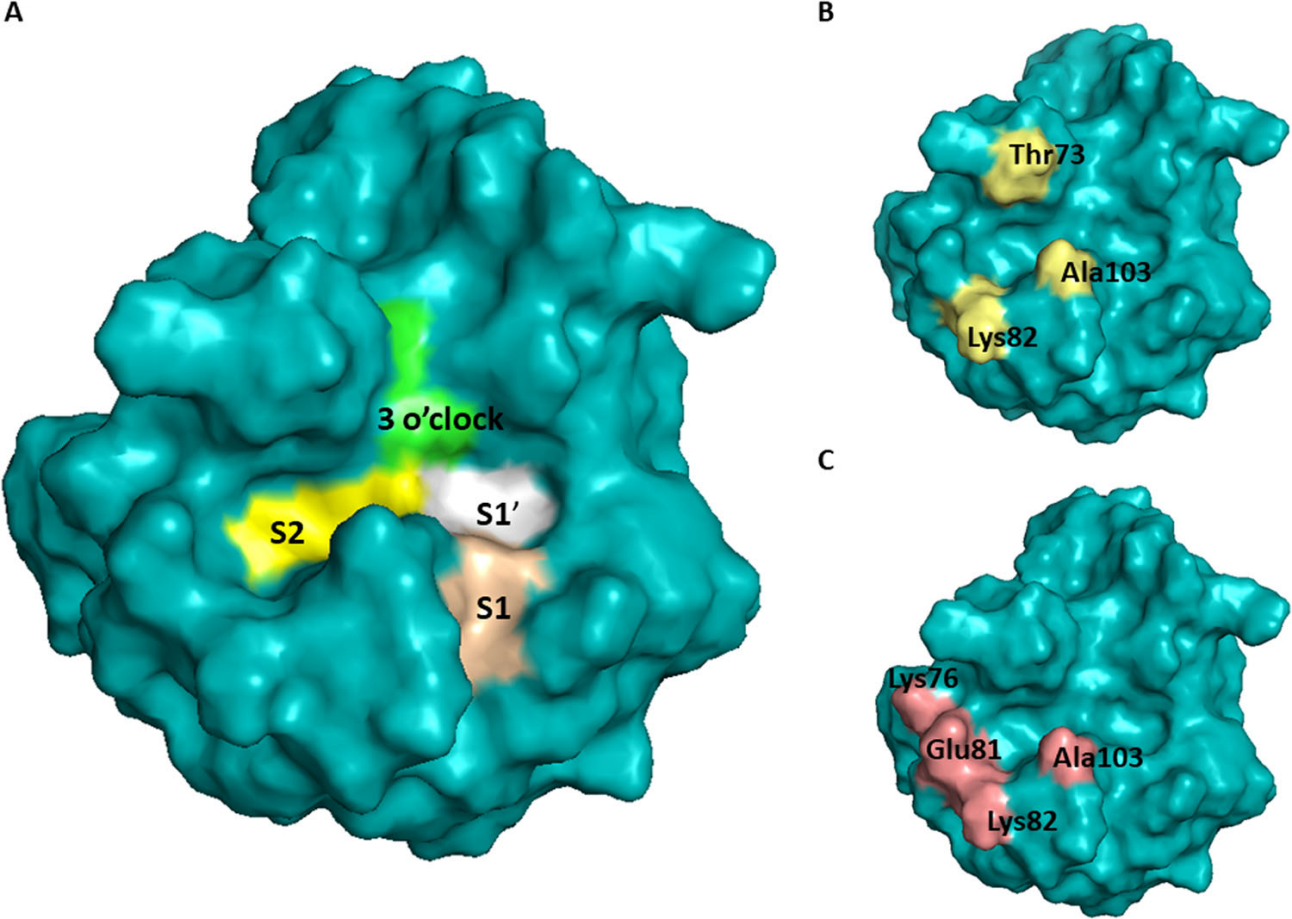
Surface representation of Cyp binding pockets and S2 gatekeepers (PDB ID: 2CPL). (A) Surface representation of binding pockets S1ʹ, S2, S1 and three o'clock pocket. (B) S2 gatekeeper residues by Davis et al. [5] (Thr73, Lys82, Ala103 of CypA). (C) S2 gatekeeper residues by Peterson et al. [181] (Lys76, Glu81, Lys82, Ala103 of CypA). Surface representation created using PyMOL software [19]. [Color figure can be viewed at wileyonlinelibrary.com]

Current research focuses on the development of selective cyclophilin inhibitors that could have the potential to treat various diseases with minimal side‐effects. For the development of selective inhibitors, it is important to know the structure of the target Cyp as well as the structural differences from the other members of Cyp family. From the information available so far, we can say that the active site of cyclophilins generally contains two main binding pockets, the catalytic S1ʹ pocket and S2 pocket [5]. The conserved catalytic S1ʹ pocket binds proline residues in proteins and catalyses their *cis*‐*trans* isomerization. The S2 pocket is probably responsible for substrate specificity, as it contains a set of residues called gatekeepers that differ among particular members of Cyp family (Figure 23) [5].

Davis et al. [5] characterized the S2 gatekeeper region as a set of residues on the surface of the pocket at position Thr73 (gatekeeper 1), Lys82 (gatekeeper 2), and Ala103 (gatekeeper 3) in CypA (Figure 23B). In silico experiment with five cyclophilin isoforms and various test peptides (general form AA_1_‐AA_2_‐Gly‐Pro, corresponding to substrate positions P3‐P2‐P1‐P1ʹ) was performed to gain knowledge about gatekeeper identity and its role in accessibility to the S2 pocket. For example, simulations for CypA showed the preference for acidic residues at the P3 position and aromatic residues at the P2 position, which was in accordance with the previous in vitro phage display experiments [379]. These results were then experimentally validated by monitoring catalysis of the peptide substrates test set and showed the importance of P3 and P2 positions in the ability of Cyps to bind and catalyse proline containing sequences. Altogether, S2 gatekeepers could interact with residues at P2 and P3 positions and thus influence the substrate specificity.

Davis et al. [5] found additional structural diversity in the β1‐β2, α1‐β3, and α2‐β8 loop regions. β1‐β2 and α2‐β8 loop regions form a contiguous surface on the back face of the cyclophilin relative to the active site. The back of the cyclophilins has previously been shown to be a mediator of protein‐protein interactions [270, 380]. Diversity in this region could indicate different interaction partners. However, the cyclophilin substrate/inhibitor selectivity is likely determined by the S2 gatekeepers rather than these regions.

In accordance with the gatekeeper hypothesis, Peterson et al. [181] suggested that S2 gatekeepers of CypD, namely Ser123 (Glu81 in CypA), Arg124 (Lys82 in CypA), and Ala145 (Ala103 in CypA) and one far S2 residue Lys118 (Lys76 in CypA), are highly diverse among cyclophilins (Figure 23C), and interactions with them could result in selective inhibition. Consequently, these assumptions were confirmed by successful development of CypD and CypE selective inhibitors (more information Chapter 3.3).

De Simone et al. [180] described so called “three o'clock” pocket located perpendicular to the axis made of S2 and S1ʹ pockets (Figure 23A). Three o'clock pocket is not targeted by CsA or peptide substrates and due to its lower conservation through cyclophilin family presents potential selectivity site. These findings were then applied for development of selective inhibitors of CypD (more information in Section 3.3).

Additionally, Shore et al. [179] mentioned so called S1 pocket as a potential diversity region in Cyps. S1 pocket is located perpendicular to the axis made of S2 and S1ʹ pocket, but in opposite direction to three o'clock pocket (Figure 23A). However, this pocket is targeted by (4*R*)‐4[(*E*)‐2‐butenyl]‐4,*N*‐dimethyl‐*L*‐threonine (Bmt) residue of pan‐cyclophilin inhibitor CsA, and thus may not be suitable selectivity site.

### 3.3 Selective Cyclophilin Inhibitors

Each Cyp is involved in different events and molecular mechanisms in a cell, and thus there is a need for inhibitors that will act selectively for a particular isoform to avoid unwanted side effects. Due to the high conservation of the cyclophilin‐like domain across the whole family of Cyps, finding selective compounds presents a challenging task. To date, only a few publications described considerably selective inhibitors of Cyps [180, 181, 336, 381].

**Figure 24:**
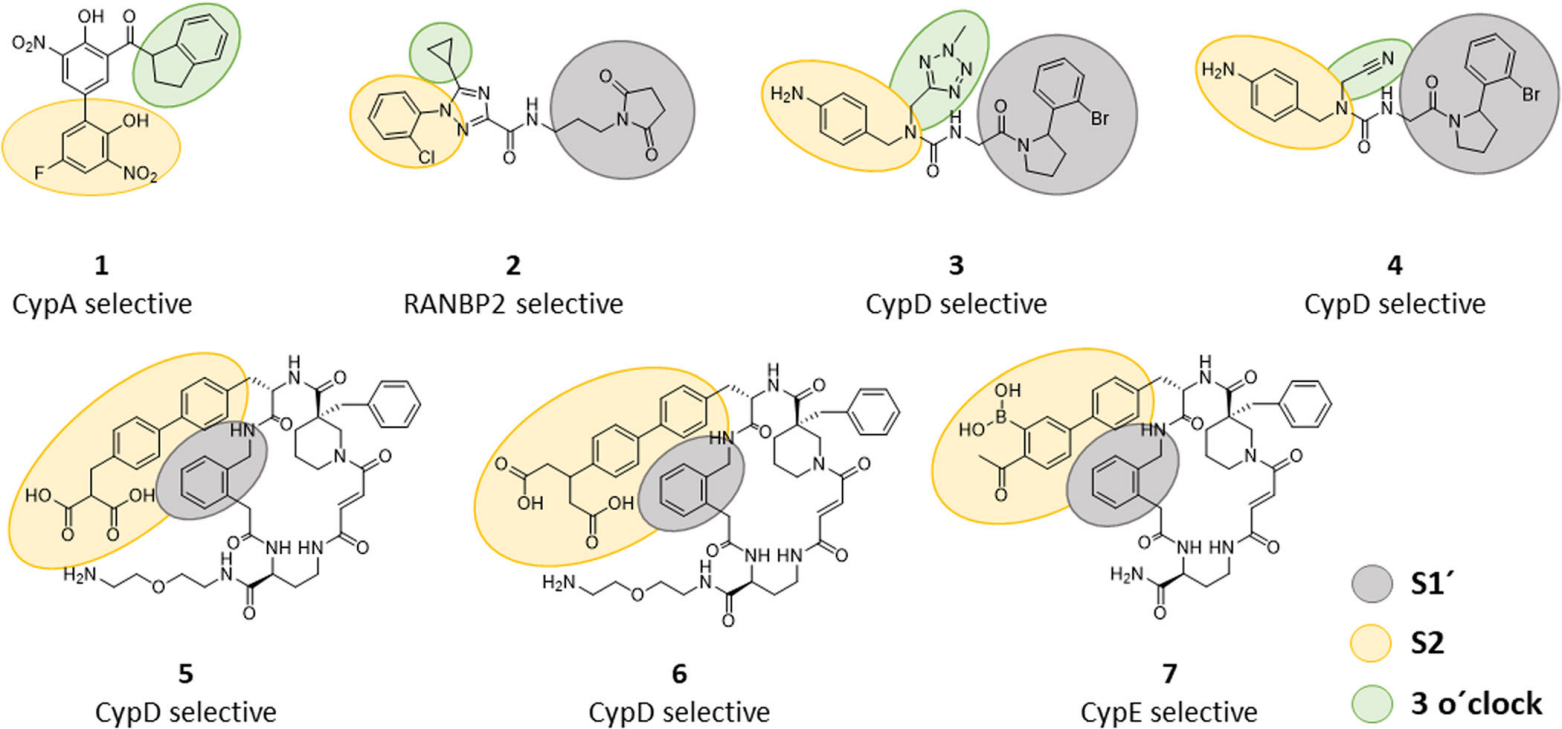
Selective cyclophilin inhibitors with depicted binding mode. Gray color indicates binding within the catalytic S1ʹ pocket, yellow and green colors indicate binding within S2 and 3 o'clock pockets, respectively. [Color figure can be viewed at wileyonlinelibrary.com]

In 2009, series of CypA selective inhibitors was developed by Daum et al. [381] The most selective inhibitor was biaryl indalyl ketone **1** (Figure 24) showing 200‐fold selectivity for CypA over CypB, CypC, and CypH, 56‐fold over PPIL1, and was also partially selective over CypD (4‐fold). Its selectivity between highly homologous CypA and CypB was surprising and without any obvious explanation [381]. A follow‐up computational study suggested, that CypB prefers a binding motif where the indanyl moiety of **1** is located in S1ʹ pocket, while CypA has the indanyl ring facing towards three o'clock pocket [382].

Later, Cho et al. [336] described compound **2** (Figure 24) as a potent and selective inhibitor of PPIase activity of RANBP2, which did not inhibit CypA. This study used virtual screening of compounds on RANBP2 followed by counter‐screening on CypA to identify selective hits. According to docking results, compound **2** binds within S1’ and S2 pockets while its cyclopropyl group orientates towards the entrance of three o'clock pocket. Whether the cyclopropyl group is responsible for the selectivity is, however, uncertain.

In 2019, De Simone at al. [180] developed a new class of tri‐vector inhibitors that bind simultaneously to S1ʹ, S2, and three o'clock pockets by redesigning existing nonselective *N*‐4‐aminobenzyl‐*Nʹ*‐(2‐(2‐ arylpyrrolidin)‐2‐oxoethyl)urea Cyp inhibitors. Alkylation of the urea nitrogen distal to the arylpyrrolidin moiety enabled targeting of the newly discovered three o'clock pocket, which was described as potential selectivity site. Indeed, several inhibitors showed certain level of selectivity toward CypD over CypA and CypB. The best inhibitor was compound **3** (Figure 24) that achieved 10‐fold selectivity over CypA and threefold selectivity over CypB. An analogous compound **4** (Figure 24) was published a year later in patent by Michel et al. [383] and showed 30‐fold and 15‐fold selectivity for CypD over CypA and CypB, respectively.

Most recently, Peterson et al. [181] designed highly potent and selective macrocyclic inhibitors of CypD and of CypE based on targeting the gatekeeper residues of their S2 pockets. The CypD selective inhibitors **5** and **6** (Figure 24) utilize hydrogen bonding between their dicarboxylate group and S2 gatekeeper residues Lys118 and Ser123 of CypD to gain selectivity over the other Cyp isoforms. Inhibitor **5** showed 100‐fold selectivity for CypD over CypA and at least 20‐fold over other tested isoforms (CypB, CypC, CypE, CypG, CypH, Cyp40, CypNK, PPIL1, PPWD1). Inhibitor **6** was slightly less potent and showed 60‐fold selectivity for CypD over CypA and at least 14‐fold selectivity over other tested isoforms. Selective inhibition of CypE by inhibitor **7** (Figure 24) was achieved through its covalent binding to the CypE residue Lys217 (Glu81 in CypA, Ser123 in CypD). Inhibitor **7** showed 230‐fold selectivity for CypE over CypA and at least 30‐fold selectivity over other tested isoforms.

## 4 Conclusion

Cyclophilins present an emerging class of drug targets. While some cyclophilins are well‐described and their physiological function as well as their role in disease development were thoroughly studied, other isoforms are only partially described and their functions remain poorly understood. The primary method of influencing cyclophilins' function involves inhibition of their PPIase enzymatic activity. Due to the high structural similarity of the active site within the cyclophilin family, achieving inhibition specific to a particular isoform poses a significant challenge. As the existing nonselective pan‐cyclophilin inhibitors are known to produce a wide range of side effects, the development of selective inhibitors will be crucial for unlocking their therapeutic potential.

## Data Availability

The data that support the findings of this study are available from the corresponding author upon reasonable request.
